# Augmenting chemotherapy with low-dose decitabine through an immune-independent mechanism

**DOI:** 10.1172/jci.insight.159419

**Published:** 2022-11-22

**Authors:** Wade R. Gutierrez, Amanda Scherer, Jeffrey D. Rytlewski, Emily A. Laverty, Alexa P. Sheehan, Gavin R. McGivney, Qierra R. Brockman, Vickie Knepper-Adrian, Grace A. Roughton, Dawn E. Quelle, David J. Gordon, Varun Monga, Rebecca D. Dodd

**Affiliations:** 1 Cancer Biology Graduate Program,; 2 Medical Scientist Training Program,; 3 Holden Comprehensive Cancer Center,; 4 Department of Internal Medicine,; 5 Molecular Medicine Graduate Program,; 6 Department of Molecular Physiology and Biophysics,; 7 Department of Neuroscience and Pharmacology,; 8 Department of Pathology, and; 9 Department of Pediatrics, University of Iowa, Iowa City, Iowa, USA.

**Keywords:** Oncology, Cancer, Drug therapy, Mouse models

## Abstract

The DNA methyltransferase inhibitor decitabine has classically been used to reactivate silenced genes and as a pretreatment for anticancer therapies. In a variation of this idea, this study explores the concept of adding low-dose decitabine (DAC) following administration of chemotherapy to bolster therapeutic efficacy. We find that addition of DAC following treatment with the chemotherapy agent gemcitabine improves survival and slows tumor growth in a mouse model of high-grade sarcoma. Unlike prior studies in epithelial tumor models, DAC did not induce a robust antitumor T cell response in sarcoma. Furthermore, DAC synergizes with gemcitabine independently of the immune system. Mechanistic analyses demonstrate that the combination therapy induces biphasic cell cycle arrest and apoptosis. Therapeutic efficacy was sequence dependent, with gemcitabine priming cells for treatment with DAC through inhibition of ribonucleotide reductase. This study identifies an apparently unique application of DAC to augment the cytotoxic effects of conventional chemotherapy in an immune-independent manner. The concepts explored in this study represent a promising paradigm for cancer treatment by augmenting chemotherapy through addition of DAC to increase tolerability and improve patient response. These findings have widespread implications for the treatment of sarcomas and other aggressive malignancies.

## Introduction

Epigenetic drugs have been of scientific and medical interest since their advent in the 1970s. DNA methyltransferase inhibitors (DNMTis) are a promising class of epigenetic modulators that demethylate DNA to reprogram cellular gene expression. Decitabine (DAC) is a deoxycytidine analogue and DNMTi that targets gene methylation through inhibition of DNA methyltransferase 1 (DNMT1). DAC was originally developed as a cytotoxic chemotherapeutic agent for delivery at maximum tolerated doses (MTDs). However, prolonged myelosuppression after DAC treatment limited its clinical applications. Recognition of DAC’s robust DNA-demethylating activity at lower doses led to its reintroduction in the clinic at approximately 5% of the original MTD (1). At these doses, DAC became a mainstay of myelodysplastic syndrome treatment regimens. More recently, interest has grown in leveraging the epigenetic effects of even lower doses of DAC to augment standard-of-care therapies for solid tumors. Studies of combination therapies using this low-dose DAC approach have shown promising results in several cancer types, including melanoma, ovarian cancer, and colorectal cancer (2–5).

Global DNA hypomethylation and reactivation of silenced antitumor genes was initially proposed as the main mechanism of action for DNMTi-based therapies. However, identification of specific gene targets and their relevance to therapeutic outcomes remains unclear, despite many efforts at genome-wide methylation profiling (6–9). Recent data from epithelial tumor models have suggested a robust role for the immune system in the antitumor activity of demethylating agents. These studies demonstrated upregulation of the viral-response pathway through expression of normally silenced endogenous retroviral (ERV) genes, resulting in increased infiltration of antitumor CD8^+^ T cells (10–13). On the basis of these findings, many groups have combined DNMTi agents with immunotherapy approaches, particularly in ovarian and colorectal cancer models (14, 15). However, few studies have combined DAC with chemotherapy for the treatment of solid tumors. Here, we explore the efficacy and elucidate the mechanism of DAC in combination with gemcitabine (Gem), a chemotherapy commonly used in the treatment of many solid tumors, including sarcoma, pancreatic, bladder, breast, ovarian, head and neck, and non–small cell lung cancers (16–20).

Sarcomas are a heterogenous group of aggressive cancers of mesenchymal origin. The outcome for patients with high-grade sarcoma has remained unchanged for decades. One of the most common forms of adult sarcoma is undifferentiated pleomorphic sarcoma (UPS), an intrinsically chemoresistant tumor that most frequently develops in the large muscles of limbs. Despite broad chemotherapy resistance, patients with advanced sarcoma are routinely treated with chemotherapy when the disease can no longer be adequately treated with surgery or radiation. The relative rarity of individual soft tissue sarcoma (STS) subtypes and widespread chemoresistance has made the study and development of novel chemotherapy regimens extremely difficult. The current standard of care for advanced STS was developed over 40 years ago. Despite a modest 26% overall response rate and 12.8-month increase in survival, a combination of doxorubicin and ifosfamide remains the most effective regimen currently available (21). In addition to having limited efficacy, doxorubicin-based regimens have a cumulative toxicity profile that limits treatment administration and decreases patients’ quality of life (22, 23). Recently, Gem-based chemotherapy has been shown to have similar efficacy as doxorubicin-based regimens but with fewer adverse side effects, making it an attractive candidate for use in new combination therapies (24).

In this study, we explore the therapeutic concept of combining DAC with the widely used chemotherapy Gem. Here, we use a combination of in vitro assays and in vivo approaches to assess fundamental aspects of the molecular and cellular events contributing to the increased efficacy of chemotherapy when combined with DAC in sarcoma. Using a well-established mouse model of aggressive, high-grade sarcoma, we demonstrate that this combination synergistically slows tumor growth and extends survival in vivo. We identify an unexpected, immune-independent mechanism by which DAC augments chemotherapy treatment in a sequence-dependent manner. The concept of leveraging sequential epigenetic therapy to improve initial chemotherapeutic response opens the door to new conceptual paradigms using DAC in the treatment of solid tumors. Furthermore, the potential of adding a well-tolerated epigenetic therapy to lower the effective dose of chemotherapy has strong implications for long-term survivorship and improved quality of life for patients with cancer.

## Results

### DAC improves chemotherapeutic response in a mouse model of high-grade sarcoma.

**Figure 1:**
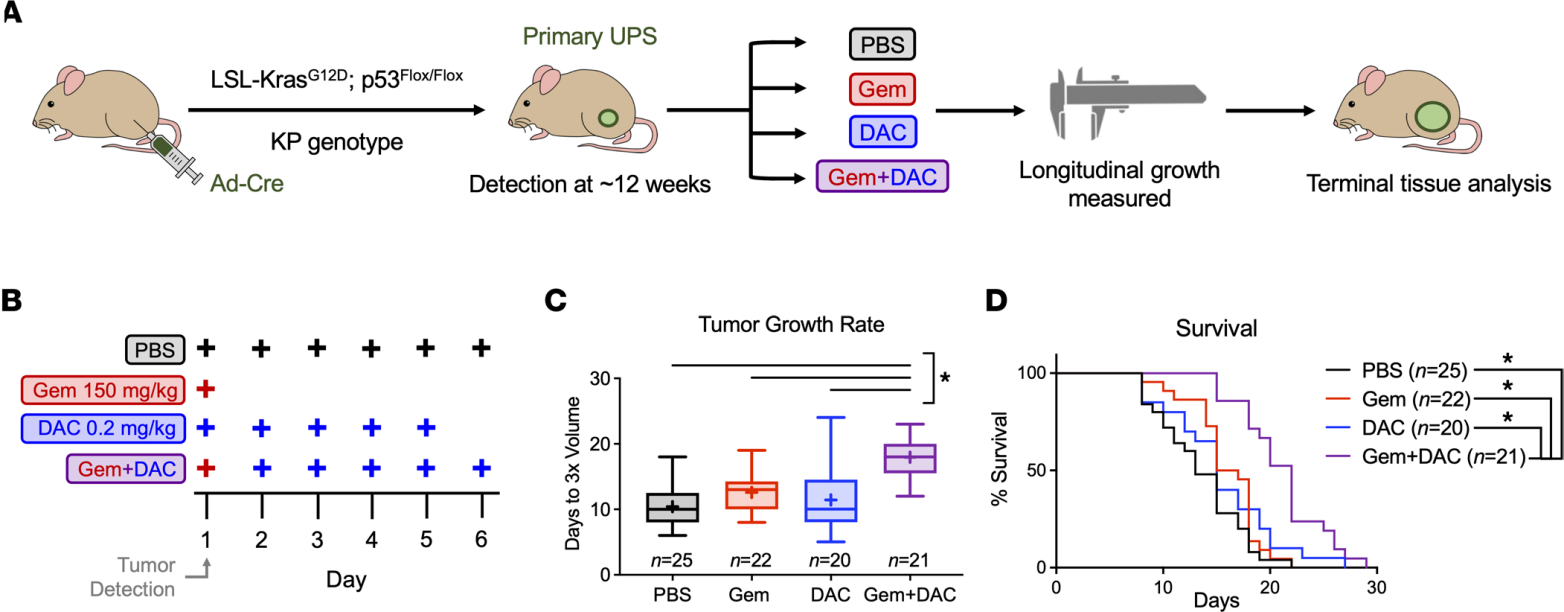
Gem + DAC slows tumor growth and extends survival in a primary mouse model of high-grade sarcoma. (**A**) KP tumors were induced in KP mice using i.m. injection of Cre recombinase adenovirus (Ad-Cre) to locally activate oncogenic Kras and delete p53. After tumor initiation, mice were randomized to 1 of 4 treatment groups: PBS, Gem, DAC, or Gem + DAC. Tumor dimensions were measured by caliper 3 times weekly, and terminal tumor tissue was collected for molecular analyses. (**B**) At the time of tumor detection, mice were placed in 1 of 4 experimental arms: 6 doses of PBS, 1 dose of Gem (150 mg/kg), 5 doses of DAC (0.2 mg/kg), or 1 dose of Gem followed by 5 doses of decitabine. (**C**) Treatment with Gem + DAC significantly slowed tumor growth compared with PBS or single-agent controls. Growth rates are reported as the time required for tumors to triple in volume (*n* = 20–25/group). Boxes represent 25th and 75th percentiles. Whiskers represent minimum and maximum values. Horizontal line represents median; + represents mean. (**D**) Treatment with Gem + DAC extended survival longer than single-agent treatments. Welch’s ANOVA and Dunnett’s T3 multiple comparison test were used to analyze the data in **C**. Log-rank (Mantel-Cox) tests with Bonferroni correction were used to analyze the data in **D**. Adjusted α = 0.00833. *P < 0.05 in **C**. *P < 0.00833 in **D**.

To examine the ability of DAC to augment chemotherapy in vivo, we used a mouse model of high-grade UPS that resembles human tumors at the molecular, pathological, and physiological levels (25–27). This model has been extensively used for preclinical studies, several of which have advanced into clinical applications (28–30). Importantly, these mice develop tumors that are surrounded by a native, immune-competent microenvironment that evolves in response to cancer growth and treatment. This approach uses Cre-loxP technology to induce tumors in the leg muscle of adult mice through localized deletion of *p53* and activation of oncogenic *Kras* by injection of an adenovirus expressing Cre recombinase into LSL-Kras^G12D^, p53^fl/fx^ (KP) mice (Figure 1A). High-grade tumors develop within 6 to 12 weeks, surrounded by an intact immune system.

After tumors reached 125 to 275 mm^3^, mice were randomized to receive PBS control, Gem alone, DAC, or Gem + DAC (dosing scheme shown in Figure 1B). No signs of toxicity were observed. Both tumor growth rate and mouse survival were used as metrics of outcome. As previously reported, this model is extremely chemoresistant (29). Treatment with Gem did not significantly extend survival compared with PBS alone (median survival of 16.0 vs 13.0 days) (Figure 1, C and D). Gem also did not slow the rate of tumor growth, with tumors tripling in volume in an average of 12.6 days compared with 10.4 with PBS control. These findings illustrate the aggressiveness of this model, which mimics the poor response of sarcoma to conventional chemotherapy regimens. Similarly, DAC monotherapy did not improve survival or slow tumor growth. DAC-treated mice displayed a median survival of 15.0 days and tumors tripled in volume in 11.4 days. In contrast, treatment with Gem + DAC significantly prolonged survival (median survival, 22.0 days) compared with PBS or single-agent treatment. Tumor growth rate was also slowed in mice treated with Gem + DAC: the time required for tumors to triple in volume was extended to an average of 18.0 days. These robust preclinical data demonstrate that addition of DAC to Gem chemotherapy significantly slows tumor growth and extends survival in an autochthonous, genetically engineered mouse model (GEMM) of high-grade sarcoma.

### Immunoprofiling reveals minimal impact of DAC on intratumoral T cell subsets.

**Figure 2:**
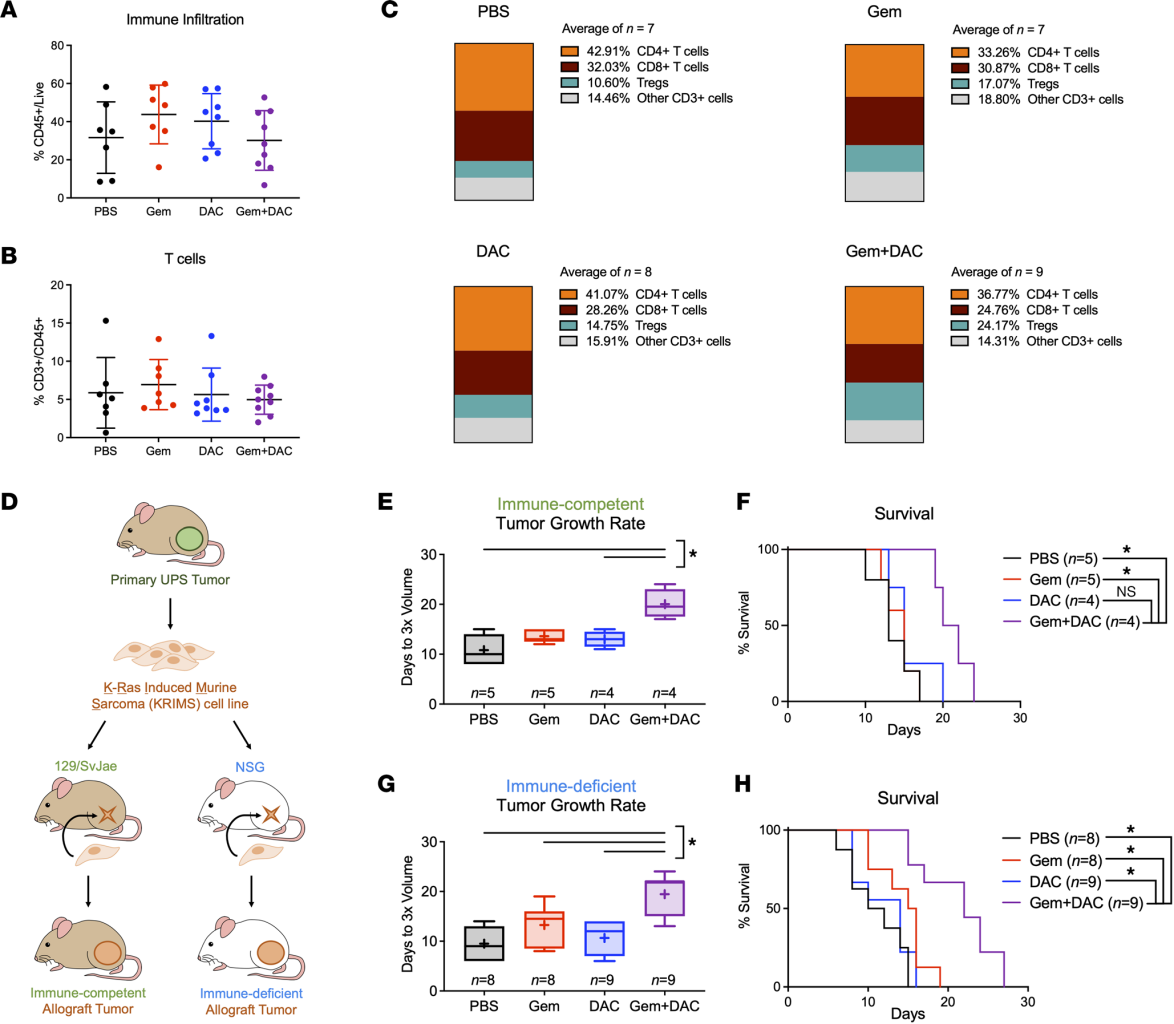
Gem + DAC efficacy is immune independent. (**A** and **B**) Tumor infiltration of total immune cells (CD45^+^) and total T cells (CD3^+^) in primary KP tumors were unchanged by Gem + DAC. Data represent individual tumors and the mean ± SD (*n* = 7–9 tumors/group). (**C**) Average frequencies of T cell populations in KP tumors, reported as percentages of total CD3^+^ T cells. Mean values are calculated from all individual tumors shown in **A** and **B**. (**D**) Generation of immune-competent and immune-deficient allograft models. KRIMS-1 cells derived from an untreated KP tumor were injected orthotopically into the gastrocnemius muscle of 129/SvJae or NSG mice. Mice were treated using the dosing scheme in Figure 1B. (**E** and **F**) Gem + DAC slowed tumor growth and prolonged survival in immune-competent 129/SvJae mice (*n* = 4–5/group). (**G** and **H**) Similarly, Gem + DAC efficacy was preserved in immune-deficient allografts in NSG mice (*n* = 8–9/group). Welch’s ANOVA and Dunnett’s T3 multiple comparison test used to analyze data in **A**, **B**, **E**, and **G**. Log-rank (Mantel-Cox) tests with Bonferroni correction were used to analyze the data in **F** and **H**. Adjusted α = 0.00833. *P < 0.05 in **E** and **G**. *P < 0.00833 in **F** and **H**.

Several reports on animal models of epithelial cancers suggest that DAC abrogates tumor growth by activating antitumor CD8^+^ T cells through a viral response pathway (10–13). We therefore hypothesized that T cell infiltration would increase in tumors treated with DAC monotherapy and the Gem + DAC combination. To determine how these therapies affect the immune microenvironment, we examined intratumoral T cell profiles by flow cytometry. We found no change in total immune infiltration between experimental arms, as shown by the pan-immune marker CD45 (Figure 2A). We also observed no changes in overall CD3^+^ T cell levels across treatment groups (Figure 2B).

Closer examination of T cell profiles in our sarcoma model revealed minimal to no affect on T cell subsets between treatment groups (Figure 2C and Supplemental Figure 1, A–D; supplemental material available online with this article; https://doi.org/10.1172/jci.insight.159419DS1). This is in striking contrast to data reported from epithelial cancer models (10–13). In our sarcoma study, CD4^+^ T helper cells comprised 33%–43% of total CD3^+^ T cells in all treatment groups. Unlike previous studies that reported DAC-induced increases in CD8^+^ T cells, we found no change in levels of cytotoxic T cells from sarcomas treated with PBS, Gem, or DAC (32.03%, 30.87%, and 28.26%, respectively). Mice receiving Gem + DAC had a slight decrease in CD8^+^ T cells (24.76%), although this was not statistically significant. Interestingly, we observed an increase in the proportion of Tregs in Gem + DAC–treated tumors compared with PBS or DAC-treated tumors (24.17% vs 10.60% or 14.75%, respectively). Of note, the effect of DAC and Gem + DAC treatment on nontumor organs, such as spleen, was also minimal (Supplemental Figure 1, E–J). Consistent with these findings, no changes in ERV gene expression or viral response–pathway transcripts were detected in terminally harvested tumors from mice receiving DAC or Gem + DAC (Supplemental Figure 2).

### Therapeutic activity of Gem + DAC is independent of T cells.

Considering the minimal changes in immune profiles observed in the Gem + DAC–treated tumors, we hypothesized that the therapeutic mechanism of Gem + DAC is immune independent. To test our hypothesis, we generated orthotopic allografts using K-ras induced murine sarcoma 1 (KRIMS-1) cells derived from a primary Kras/p53–mutant UPS tumor. We previously showed that syngeneic KRIMS-1 allografts have similar growth rates, survival, and immune infiltration as the primary KP tumors (31). To test the role of the immune system in Gem + DAC response in vivo, we injected KRIMS-1 cells orthotopically into immune-competent (129/SvJae) or immune-deficient (i.e., NOD/SCID/γ [NSG]) mice (Figure 2D). Results with the immune-competent allograft model closely match the data obtained in the primary GEMM examined in Figure 1 (Figure 2, E and F). Immune-competent tumors in 129/SvJae mice treated with Gem + DAC tripled in volume in approximately 20.0 days, and PBS, Gem-, and DAC-treated tumors tripled within 10.8, 13.6, and 13.0 days, respectively. Gem + DAC also significantly prolonged survival (median survival, 21.0 days) compared with PBS, Gem, or DAC controls (median survival, 13.0, 15.0, and 15.0 days, respectively). In immune-deficient NSG mice, Gem + DAC activity was preserved, despite these mice lacking mature T cells, B cells, and NK cells, and having defective myeloid populations (Figure 2, G and H). Gem + DAC–treated, immune-deficient tumors tripled in 19.4 days, compared with 10–13 days for PBS and monotherapy-treated tumors. Similarly, median survival was extended to 22.0 days in mice receiving Gem + DAC compared with 11.0, 15.5, and 14.0 days for mice receiving PBS, Gem, or DAC, respectively. These findings demonstrate that an intact immune system is not necessary for Gem + DAC activity in vivo and suggest that an alternative, immune-independent mechanism is responsible for the therapeutic benefit observed in these mice.

### Drug sequence is critical for synergistic Gem + DAC activity in vitro.

**Figure 3:**
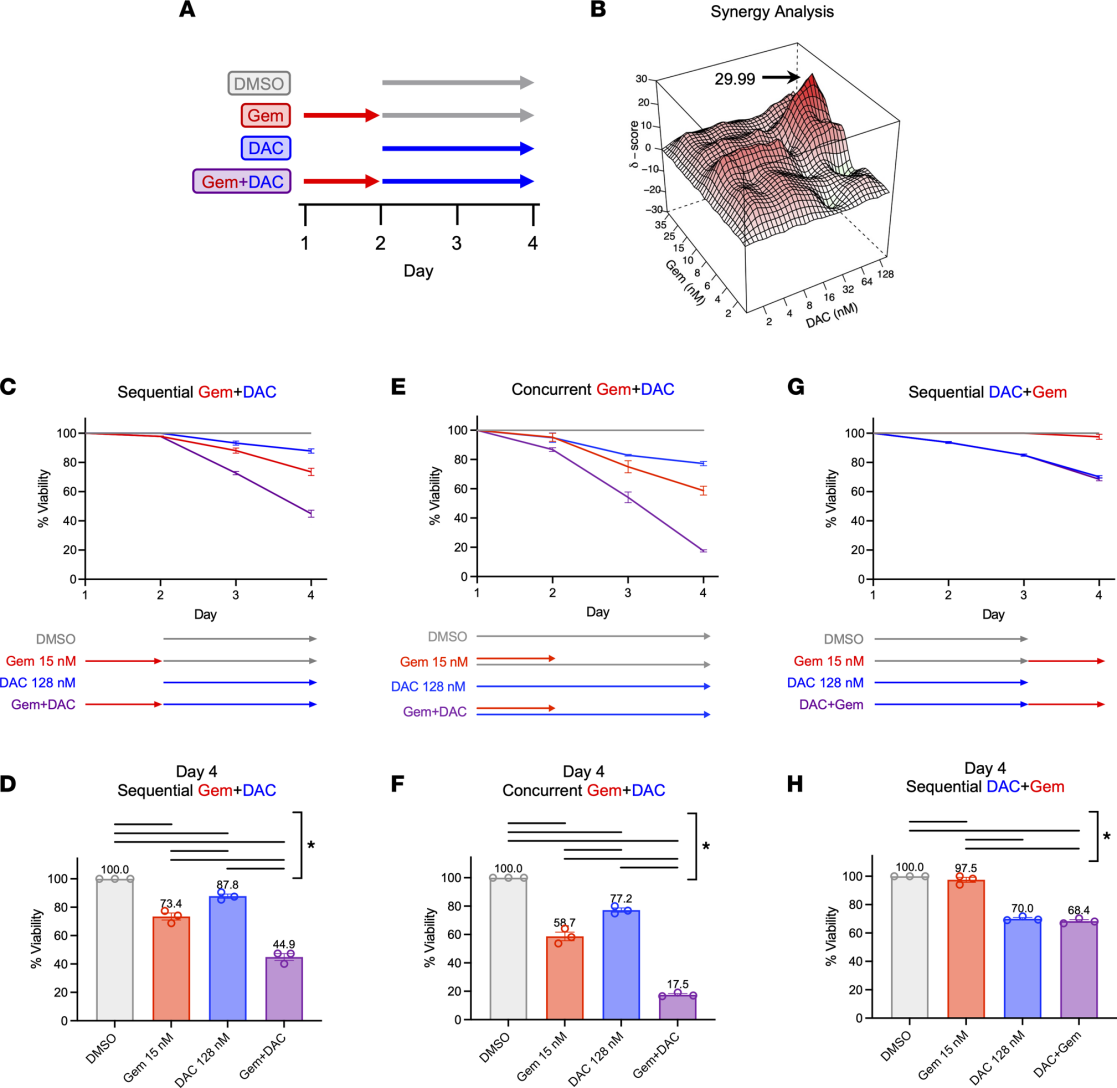
Drug sequencing is critical for Gem + DAC efficacy. (**A**) In vitro treatment scheme. KRIMS-1 cells were treated with Gem or media control on day 1, followed by DAC or DMSO control on days 2-3. (**B**) Representative synergy plot of Gem + DAC identifying concentrations that synergistically inhibit cell growth (maximum Bliss synergy score of 29.99 for gemcitabine 15 nM and DAC 128 nM). (**C–H**) Longitudinal viability and day 4 measurements of KRIMS-1 cells using different sequences of delivery for Gem (15 nM) and DAC (128 nM). (**C** and **D**) Sequential administration of Gem followed by DAC. (**E** and **F**) Concurrent administration of Gem + DAC treatment. (**G** and **H**) Reversed-sequence DAC + Gem treatment, with DAC preceding Gem treatment. Individual viability measurements and statistical analysis for data in **C**, **E**, and **G** are available in Supplemental Figure 8. For **C**–**H**, data represent independent experiments (*n* = 3) and the mean ± SEM. Ordinary 1-way ANOVA and Tukey’s multiple comparisons test used to analyze data in **C**–**H**. *P < 0.05.

To explore the mechanisms driving the activity of Gem + DAC, we treated KRIMS-1 cells in vitro with a similar dosing strategy used for in vivo studies described above (Figure 3A). Dose-response curves revealed KRIMS-1 cells are moderately sensitive to Gem, with IC_50_ values in the nanomolar range. In contrast, these cells are resistant to DAC, with IC_50_ values approaching micromolar levels (Supplemental Figure 3 and Supplemental Figure 4). Using the Bliss independence model to assess drug synergy (32, 33), we investigated increasing concentrations of Gem and DAC after 4 days of incubation (Figure 3B). Gem + DAC treatment generally was additive (δ score, 0–10), with a strong synergistic interaction (δ score, >10) occurring with 15 nM Gem and 128 nM DAC (δ score, 29.99). The combination of 15 nM Gem and 128 nM DAC was identified as being strongly synergistic, using 3 different synergy analyses: Bliss independence, highest single agent (34), and zero-interaction potency (35) (Supplemental Figure 5, A–D). Similarly, analysis of human sarcoma and carcinoma cell lines identified areas of synergistic interaction with Gem + DAC treatment, particularly in the embryonal rhabdomyosarcoma line RD, the alveolar rhabdomyosarcoma cell line SJRH30, and the pancreatic ductal adenocarcinoma cell line MIA PaCa-2 (Supplemental Figures 3, 4, 6, and 7).

We next assessed, through a series of longitudinal studies, how the order of drug delivery influenced these synergistic effects. First, we tested sequential Gem + DAC and observed decreased viability at days 3 and 4 in cells receiving the combination treatment compared with monotherapy (Figure 3, C and D; Supplemental Figure 8A; and Supplemental Table 1). These data further support our finding of synergy identified in Figure 3B. Gem and DAC monotherapies reduced viability to 73.4% and 87.7%, respectively, compared with DMSO control. Sequential treatment with Gem + DAC reduced viability to 44.9%. We then tested the effects of concurrent Gem + DAC treatment, because coadministration of drugs is a more logistically feasible treatment scheme to use in the clinic (Figure 3, E and F; Supplemental Figure 8B; and Supplemental Table 1). The synergistic effects of Gem + DAC were heightened with concurrent administration: viability was reduced to 17.5%. Finally, we tested the reverse order of drug delivery by treating first with DAC on days 1 and 2, followed by Gem on day 3 (Figure 3, G and H, Supplemental Figure 8C, and Supplemental Table 1). Unlike the sequential and concurrent Gem + DAC treatments, DAC + Gem (i.e., DAC given first) only reduced viability to 68% and did not perform better than DAC alone. Similar results were seen in vivo, with the Gem + DAC sequence having a stronger impact on overall survival and tumor growth compared with DAC + Gem (Supplemental Figure 9, A–C). These in vitro and in vivo findings suggest that the synergistic interaction of Gem + DAC is sequence dependent, relying on the initial presence of Gem to modulate response to DAC.

### Gem + DAC treatment induces apoptosis and cell cycle arrest.

**Figure 4:**
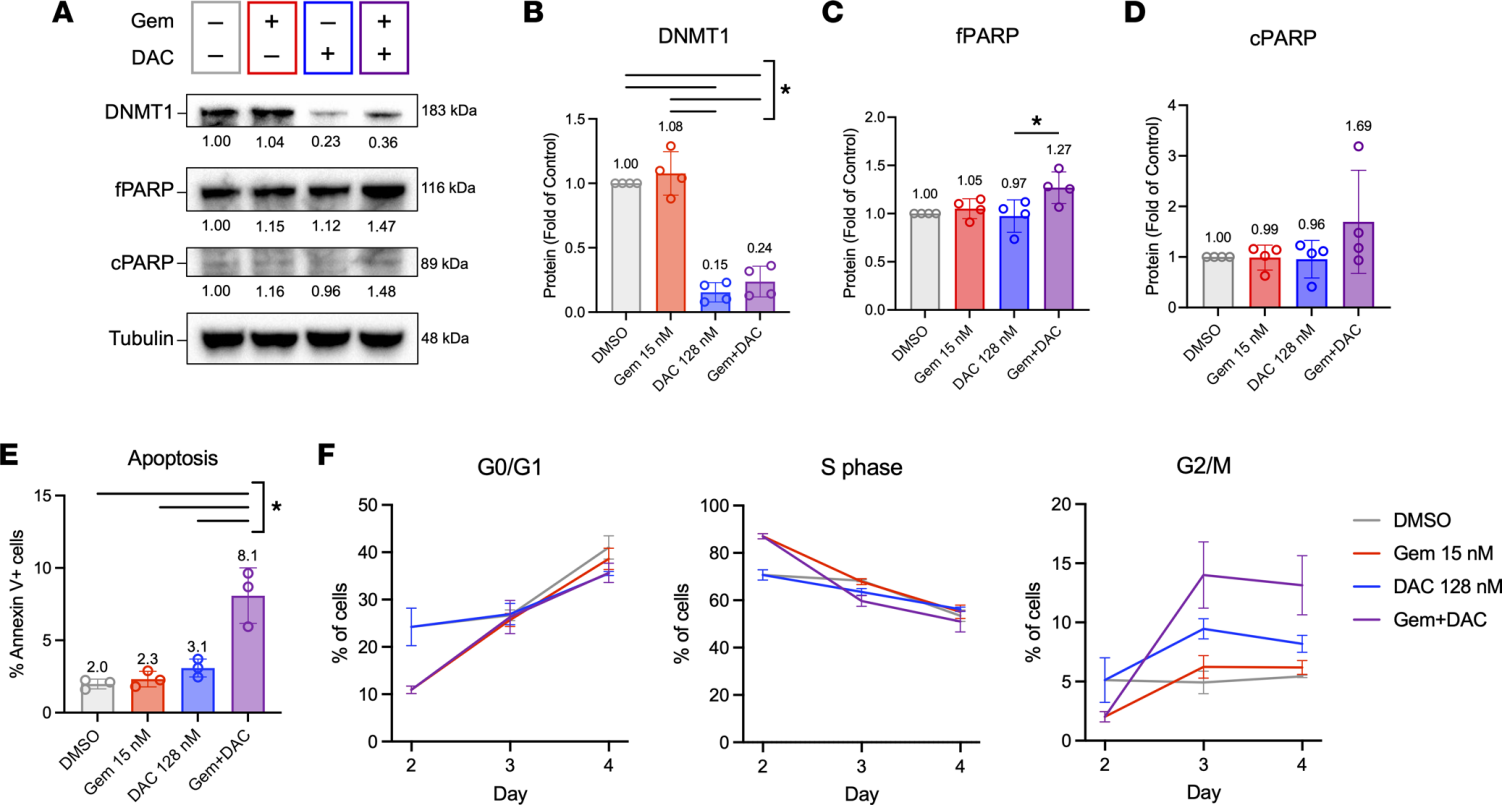
Gem + DAC induces apoptosis and cell cycle arrest. (**A**–**D**) Representative Western blot and quantification of lysates collected on day 4. Data represent independent experiments (*n* = 4) and the mean ± SD. (**B**) Levels of DNMT1 are decreased in cells treated with DAC and Gem + DAC. (**C** and **D**) Levels of full-length PARP (fPARP) and cleaved PARP (cPARP) are not altered across treatments. (**E**) Day 4 measurement of annexin V staining shows increased apoptosis in Gem + DAC–treated cells. (**F**) Longitudinal cell cycle analysis using EdU/PI staining shows increased cell cycle arrest and accumulation in G2/M in Gem + DAC–treated cells. Complete statistical analysis is available in Supplemental Figure 10. Data in **E** and **F** represent independent experiments (*n* = 3) and the mean ± SD. Ordinary 1-way ANOVA and Tukey’s multiple comparisons test were used for analysis of data in **B**–**F**. *P < 0.05.

We next explored the mechanisms by which Gem + DAC reduces cell growth. We examined multiple mechanisms that could be responsible for reduced cellular viability, including markers of DNA damage, apoptosis, senescence, and cell cycle arrest. As expected, DAC monotherapy and Gem + DAC decreased DNMT1 protein levels and 5-methylcytosine levels in genomic DNA, demonstrating that canonical DAC activity is preserved with Gem + DAC treatment (Figure 4, A and B, and Supplemental Figure 10A). Both DAC monotherapy and Gem + DAC reduced DNMT1 and 5-methylcytosine levels to a similar extent, indicating that Gem + DAC efficacy is not primarily driven by decreased levels of DNMT1 or DNA hypomethylation. Immunoblot studies further demonstrated that Gem + DAC treatment does not appreciably alter levels of full-length PARP or cleaved PARP, which are markers of DNA damage and apoptosis, respectively (Figure 4, C and D). Similarly, levels of γH2AX, a marker of DNA double-strand breaks, were only modestly increased by Gem + DAC treatment (Supplemental Figure 10B).

To directly measure the impact of Gem + DAC on apoptosis, we performed annexin V staining. Using this method, we observed that Gem + DAC induced apoptosis in 8.1% of cells, compared with 2.0%–3.1% in control or monotherapy-treated cells (Figure 4E). Despite the significant increase in the number of apoptotic cells, the relatively low magnitude of apoptosis in the Gem + DAC population could not account for the large decreases in viable cells identified in Figure 3. Next, using analysis of cell morphology by brightfield microscopy, we examined potential induction of cellular senescence (Supplemental Figure 11, A–E). Some cell enlargement and flattening, features classically associated with senescence, were observed with DAC and Gem + DAC treatments. However, these changes were minimal compared with the effects of strongly prosenescent MEK and CDK4/6 inhibitor therapies (36).

Finally, we assessed the impact of Gem + DAC on cell cycle progression during the 4-day treatment. Longitudinal cell cycle analysis revealed that in addition to promoting apoptosis, Gem + DAC caused cell cycle arrest (Figure 4F and Supplemental Figure 10, C–E). The effects of Gem + DAC on cell cycle progression varied over time. A significant 16.3% increase in S-phase arrest was detected at day 2 of treatment, as expected from Gem on the basis of previous literature (37). After removing Gem and adding DAC, an increase in G2/M arrest was observed on days 3 and 4. Gem + DAC caused greater than a 2-fold increase in G2/M compared with vehicle and Gem controls, and a 1.6-fold increase compared with single-agent DAC. These results strongly suggest that a key feature of the Gem + DAC combination therapy is slowed proliferation due to sustained cell cycle arrest.

### Decreased cellular deoxycytidine levels drive Gem + DAC activity.

**Figure 5:**
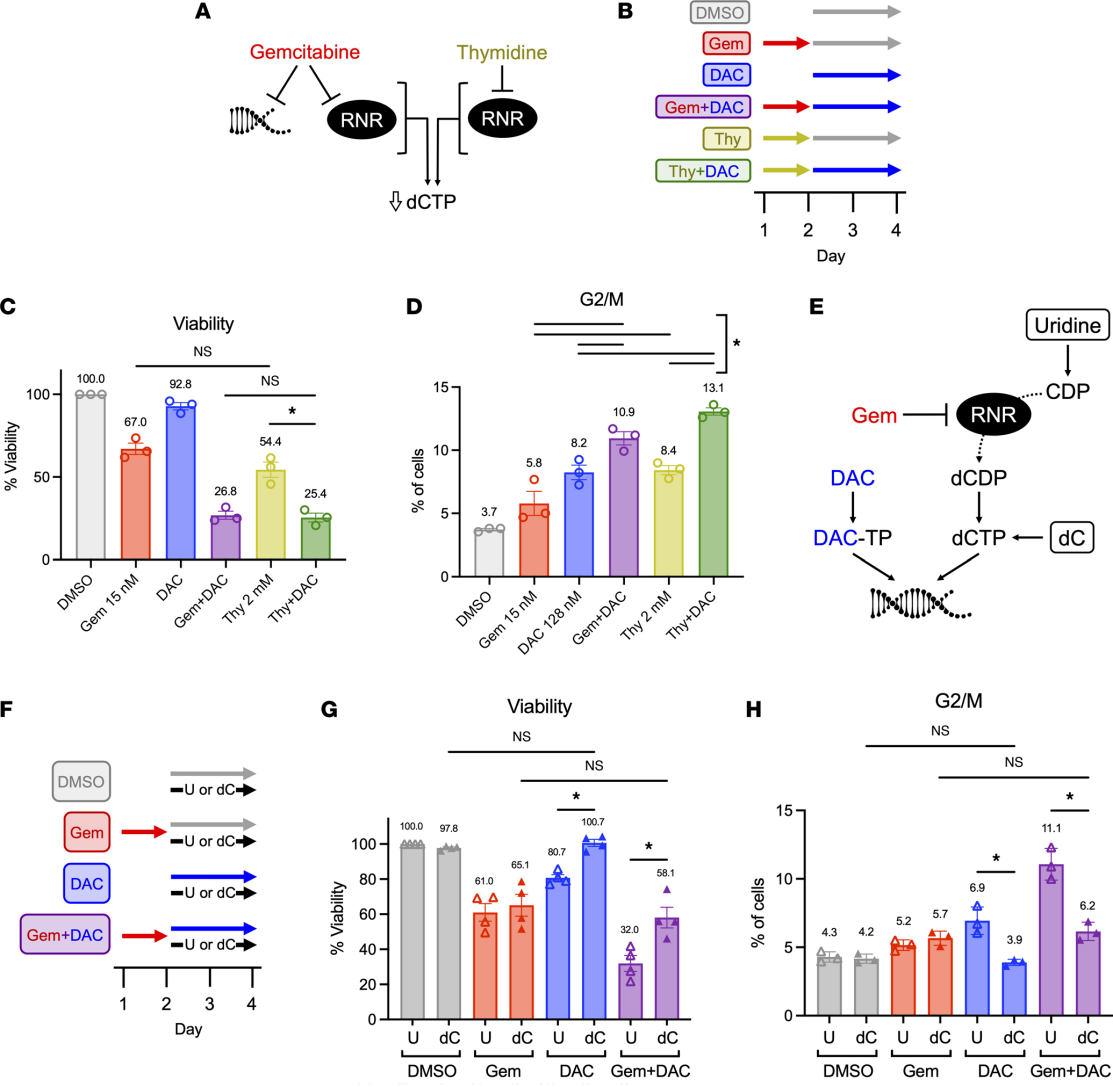
Decreased cellular dCTP drives increased efficacy of Gem + DAC. (**A**) Gem causes direct termination of DNA polymerization and irreversible inhibition of RNR, resulting in decreased levels of dCTP. Thy inhibits RNR and causes a similar decrease in dCTP levels. (**B**) Treatment schematic for Gem + DAC and Thy + DAC single-agent and combination approaches. (**C**) At day 4, Thy and Thy + DAC decrease viability to the same extent as Gem and Gem + DAC, respectively. (**D**) Day 4 G2/M analysis using EdU/PI staining shows similar cell cycle arrest in Thy-containing treatments. Complete statistical analysis of **C** and **D** is available in Supplemental Figure 12. (**E**) Gem inhibits RNR, decreasing cellular dCTP and increasing the ratio of DAC to dCTP. Levels of dCTP can be directly augmented by addition of exogenous deoxycytidine (dC), but not uridine (U), due to inhibition of RNR by Gem. (**F**) Uridine (30 μM) or dC (30 μM) was added during DAC or DMSO treatment on days 2 and 3. (**G** and **H**) Addition of dC, but not uridine, rescued the effects of DAC on viability and G2/M arrest in cells treated with both DAC and Gem + DAC treatments. Complete statistical analysis of the data in **G** and **H** is available in Supplemental Figures 13 and 14. Data in **C**, **D**, **G**, and **H** represent independent experiments (*n* = 3) and the mean ± SEM. Ordinary 1-way ANOVA and Tukey’s multiple comparisons test used for analysis. *P < 0.05.

Gem, a deoxycytidine analogue (2′,2′-difluoro-2′-deoxycytidine), induces cytotoxic effects through 2 well-established mechanisms: direct termination of DNA polymerization and irreversible inhibition of ribonucleotide reductase (RNR) (Figure 5A). Gem-induced RNR inhibition decreases cellular deoxyribonucleotide pools, particularly 2′-deoxycytidine 5′-triphosphate (dCTP) levels (38). The decrease in cellular dCTP potentiates the effects of Gem by decreasing its competition with dCTP for incorporation into DNA (39). Like Gem, DAC is a deoxycytidine analogue (5-aza-2′-deoxycytidine). Inhibition of RNR augments DAC efficacy and increases its incorporation into DNA (40). On the basis of these known mechanisms of action and our data demonstrating the importance of drug sequencing and cell cycle inhibition (Figures 3 and 4), we hypothesized that Gem + DAC activity is based on initial inhibition of RNR by Gem, which primes cells for treatment with DAC. We further predicted that DAC activity is more effective in combination-treated cells because of an increased DAC to dCTP ratio, resulting in elevated and sustained cell cycle arrest. To test the first part of this hypothesis, we blocked RNR activity with an alternative compound, substituting thymidine (Thy) for Gem in the sequential Gem + DAC dosing strategy (Figure 5B). Like Gem, Thy is a potent inhibitor of RNR that specifically depletes dCTP levels (41, 42). However, in contrast to Gem activity, Thy does not directly inhibit DNA polymerization (Figure 5A), making it a useful tool compound for dissecting the mechanistic activity of Gem in Gem + DAC therapy.

As expected, Thy treatment decreased cell viability to a similar extent as Gem (Figure 5C and Supplemental Figure 12A). In combination with DAC, Thy has similar effects as Gem, with Thy + DAC treatments reducing cellular viability to 25.4%, which is nearly identical to the 26.8% viability observed after Gem + DAC treatment. Similarly, Thy + DAC also alters cell cycle progression by promoting G2/M arrest, as seen with Gem + DAC treatment (Figure 5D and Supplemental Figure 12, B–D). Approximately 8% of cells were in G2/M arrest after treatment with Thy or DAC alone. This increased to 13.1% with Thy + DAC, similar to 10.9% with the Gem + DAC combination. Although Thy had a greater impact on G2/M arrest than did Gem (8.4% vs 5.8%), this ~2% difference persisted when combined with DAC, suggesting that the magnitude of DAC’s impact is similar in Gem + DAC and Thy + DAC treatments. These data demonstrate that blocking RNR with a tool compound prior to DAC treatment has similar effects to Gem + DAC on cell viability and G2/M arrest, supporting the conclusion that Gem primes cells for DAC activity through inhibition of RNR.

As a deoxycytidine analogue, DAC must compete with the intracellular dCTP pool for incorporation into DNA. Because both Gem and Thy specifically decrease cellular dCTP levels, the second part of our hypothesis predicted that the augmented effectiveness of Gem + DAC therapy results from an elevated DAC to dCTP ratio after Gem treatment. To test that idea, we treated KRIMS-1 cells with sequential Gem + DAC in the presence of supplemental deoxycytidine or uridine during DAC treatment (Figure 5F). Deoxycytidine supplementation has been shown to rescue dCTP levels after RNR inhibition (42), to decrease DAC’s incorporation into DNA (43), and to decrease DAC’s therapeutic efficacy (40). Uridine, in contrast, must be processed through the nucleotide salvage pathway and RNR before it can be incorporated into DNA as dCTP (Figure 5E). As predicted, supplementation with deoxycytidine after initial Gem treatment did not alter the effects of Gem compared with uridine control (Figure 5G and Supplemental Figure 13, A–D). In contrast, deoxycytidine completely protected cells from DAC monotherapy. The presence of deoxycytidine also significantly decreased the impact of Gem + DAC on cell viability. Gem + DAC with deoxycytidine reduced viability only to 58.1%, whereas Gem + DAC with uridine reduced viability to 32.0%. Effects on cell cycle progression further supported this hypothesis (Figure 5H and Supplemental Figure 14, A–C). As with cell viability, deoxycytidine supplementation reduced G2/M arrest in Gem + DAC–treated cells to similar levels seen with Gem. These data suggest that Gem potentiates the efficacy of DAC by increasing the DAC to dCTP ratio. Biologically, the relative increase in cellular DAC synergizes with Gem to induce both apoptotic cell death as well as G2/M–phase cell cycle arrest.

## Discussion

Epigenetic drugs such as DAC act as genetic modulators in a variety of tissues and are attractive candidates for combination therapies that sensitize tumors to conventional therapies. Most prior studies focused on using DAC as a pretreatment to sensitize tumor cells to immunotherapy (44). However, the ability of DAC to augment chemotherapy in solid tumors has not been extensively studied, and the few studies that have been conducted used DAC as a chemotherapy pretreatment. In this study, we investigated the efficacy and mechanism of a sequential combination therapy consisting of Gem followed by DAC in a GEMM of adult STS.

Our data show that treatment with Gem + DAC therapy significantly improves survival and slows tumor growth in these high-grade tumors. Unlike prior reports using epithelial tumor models, this effect was immune independent. Gem + DAC had minimal impact on intratumoral T cell subsets and in vivo efficacy was maintained in an immune-deficient mouse model. Further analysis demonstrated a critical role for the order of drug delivery, with reversal of the drug sequence completely abolishing all synergistic effects. This led us to uncover a unique mechanism for augmenting the effects of chemotherapy. Our data showed that Gem primes cells for treatment with DAC through inhibition of RNR. Subsequent treatment with DAC enhances and sustains Gem-initiated apoptosis and cell cycle arrest to synergistically reduce tumor cell growth. The concepts explored in this study may represent a new paradigm in augmenting chemotherapy by addition of DAC. The ability of sequential DAC treatment to lower toxicities and improve response in chemoresistant tumors could have widespread implications for cancer treatment.

Few studies have combined DAC with chemotherapy, and those that have almost exclusively used DAC as a pretreatment to sensitize tumors to chemotherapy. Here, we demonstrate that DAC can synergize with chemotherapy when administered as the second drug in a sequence. To our knowledge, this is the first study in which DAC was used to sequentially augment initial chemotherapy treatment in a solid tumor model. Such a regimen can easily be translated to clinic with DAC given after the administration of standard-of-care chemotherapy. Additionally, our data demonstrate that concurrent Gem + DAC treatment retains its synergistic efficacy. Concurrent combination therapy is easier to administer in the clinic and benefits patients, because it reduces the number of times they must travel for treatment. Indeed, encouraging results from a recent phase I clinical trial (ClinicalTrials.gov NCT02959164) in advanced adult sarcoma patients have shown that codelivery of DAC with Gem is well tolerated and can improve outcomes in some adult sarcomas (45). Importantly, our finding that the reversal of treatment order (DAC + Gem) results in loss of synergistic effects highlights the fact that not all tumors can be effectively sensitized by DAC. Furthermore, those that are resistant to sensitization may still respond to DAC if it is administered using a different treatment regimen.

This work sheds new mechanistic insight into how DAC can synergize with Gem, which has important implications for the development of future drug combinations with this approach. Through our studies, we determined that Gem + DAC induces a biphasic cell cycle arrest. Initial Gem-driven RNR inhibition bolsters the efficacy of DAC, possibly by decreasing its competition with endogenous dCTP at becoming incorporated into DNA. Gem + DAC combination therapy may be a promising option for patients who are routinely treated with Gem as standard of care, such as those with pancreatic cancer. Extending from our data, future investigations could assess other drugs that target nucleoside metabolism in combination with DAC to bolster therapeutic efficacy. For example, RNR-inhibiting drugs such as clofarabine and hydroxyurea are already approved for clinical use, and several others, including triapine and TAS1553, have shown promise in preclinical and early clinical trial studies (ClinicalTrials.gov NCT02466971 and NCT04637009) (46). The clinical implications for sequential RNR inhibition and low-dose nucleoside analogue administration are widespread and promising.

In our murine sarcoma models, we observed that DAC does not induce an antitumor CD8^+^ T cell response, nor does Gem + DAC require an intact immune system to slow tumor growth. These data are in contrast to other in vivo studies in epithelial cancer models that identify strong immunomodulatory effects after treatment with DAC (10–13). In these studies, other groups observed that DAC increases antitumor immune activation through expression of previously methylated ERV genes. Hypomethylation of ERV genes in tumor cells can result in transcription and formation of dsRNA that are sensed by cytosolic pathogen-sensing, pattern-recognition receptors such as MDA5/MAVS. This leads to production and release of type 1 IFNs that act in an autocrine feedback loop to stimulate tumor cell production of chemokines such as CXCL9/10 and expression of other pro-inflammatory genes. This results in increased CD8^+^ T cell infiltration and priming of the tumor for treatment with immune checkpoint blockade therapy. Importantly, DAC-induced antitumor immune activation depends on the ability of a tumor cell to initiate a robust immunostimulatory signaling cascade in its microenvironment and on the ability of the immune system to respond to this pro-inflammatory signaling. In disease models lacking tumor cells, DAC has direct inhibitory effects on the immune system (47–54), including the induction of Tregs and suppression of γδ T cells.

We hypothesize that the lack of immune stimulation by DAC in our mouse model can be partially explained by the low levels of immune infiltration in the sarcoma tumor microenvironment. Human sarcomas are generally considered immunologically “cold” and contain lower levels of intratumoral lymphocytes than epithelial-derived malignancies such as breast cancer, colon cancer, or renal cell carcinoma (55). They also have low mutational burdens (56), and their intratumoral immune landscape is dominated by tumor-associated macrophages (57). Multiple studies have demonstrated the ineffectiveness of immunotherapies in treating a variety of sarcoma subtypes (58–62), further supporting the idea that tumors of mesenchymal origin contain unique barriers to immunomodulation. Though the immunostimulatory effects of DAC were generally absent in our sarcoma mouse model, the immune-independent synergy of Gem + DAC is encouraging for its use in the clinic. As with many other cancers, sarcomas are commonly treated with aggressive radiation and chemotherapy regimens that leave patients severely immunocompromised. The therapeutic mechanism of Gem + DAC would be preserved in these and other immunocompromised patients, suggesting this approach can be a valuable and effective tool during multiple phases of cancer treatment.

DAC was originally engineered as a chemotherapeutic nucleoside analogue for administration at MTDs to achieve cytotoxicity. More recent applications have taken advantage of its role as a DNMT1 inhibitor and used DAC to induce epigenetic changes including reactivation of methylated tumor suppressor genes and activation of antitumor immunity. Our data uncover another application of DAC that can augment the cytotoxic effects of conventional chemotherapy. We used complementary in vitro assays and in vivo approaches to demonstrate the ability of DAC to ultimately augment both the cytotoxic and cytostatic effects of conventional chemotherapy. This approach reduces the amount of harmful chemotherapy needed to achieve a robust reduction in tumor cell growth by supplementing standard chemotherapy with low, less-toxic doses of DAC. These results have strong implications for future applications using DAC to increase the efficacy and tolerability of conventional chemotherapies in multiple cancers.

## Methods

### Mice.

All animal experiments were performed in accordance with protocols approved by the IACUC at the University of Iowa. The KP sarcoma model has been previously described (25–27, 31). To induce UPS formation in KP mice, 25 μL of Ad-Cre (University of Iowa Viral Vector Core, Iowa City, IA) was mixed with 3 μL of calcium chloride (2 M) and 600 μL of DMEM (Gibco, 11965-092). Following a 15-minute incubation at room temperature, 50 μL of the mixture was injected into the gastrocnemius muscle. As previously published (31), orthotopic allografts were generated using the KRIMS-1 cell line. Trypsinized cells were washed and resuspended in sterile PBS containing calcium chloride and magnesium chloride. All cells were approximately 90% confluent on the day of injection. Mice maintained on a 129/SvJae background or NSG background were injected with 50 μL of cell suspension containing 2.5 × 10^5^ cells in the left gastrocnemius muscle using a 31G needle. For both primary and allograft models, day 1 of tumor growth was defined when sarcomas first reach a volume of 125 to 275 mm^3^. Starting on day 1, mice were treated with PBS, Gem hydrochloride 150 mg/kg (MedChemExpress, HY-B0003; Sigma-Aldrich, 504594) diluted in water, DAC 0.2 mg/kg (Selleckchem, S1200) diluted first in DMSO to 1 mg/mL and then in PBS to 0.035 mg/mL, or a combination of the 2 using the dosing scheme in Figure 1B. Tumors were measured by digital caliper 3 times weekly, and volume was calculated using the formula V = (π × L × W × H)/6, with L, W, and H representing the length, width, and height of the tumor in mm, respectively. Tumors outside of specified volume range at the time of detection were excluded from the study. Terminal tumor volume was set at 1000 mm^3^. All mouse strains were maintained in author RDD’s laboratory colony. Male and female mice older than 7 weeks were used for all studies.

### Flow cytometry profiling of immune subsets.

As previously published (31), terminally harvested tumors were washed with 5 mL of PBS in a 6-well plate and finely minced with surgical scissors. To each well containing tumor tissue, we added 4.5 mL of Collagenase Type IV (700 units/mL, Gibco, 17104-019) and 0.5 mL of FBS. Plates were incubated for 1 hour at 37°C on an orbital shaker. After incubation, dissociated tissue was passed through a 70 μM cell strainer into a 50 mL conical vial using a 10 mL serological pipette and the plunger from a 1 mL syringe. Cell strainers were washed with 25 mL of PBS into corresponding conical vials. Spleens were similarly harvested and minced, then immediately passed through a 70 μM cell strainer into a 50 mL conical vial using a 10 mL serological pipette and the plunger from a 1 mL syringe. Next, cell suspensions (tumor or spleen) were centrifuged, and cell pellets were resuspended in 2 mL of ACK lysis buffer (Gibco, A1049201). After 5 minutes, 10 mL of PBS were added, and samples transferred to 15 mL conical tubes and centrifuged at 500*g* for 5 minutes. Cell pellets were resuspended in cell-staining buffer (Biolegend, 420201). In a round-bottom, 96-well plate, 50 μL aliquots of cell suspensions were incubated with Zombie Aqua Viability Dye (Biolegend, 77143) and anti-CD16/32 (clone 93, Biolegend) to block Fc receptors on ice. After a 10-minute incubation, 50 μL of Abs were added and incubated on ice for 30 minutes. Abs used were anti–CD45 BV605 (clone 30-F11, Biolegend), anti–CD11b PE (clone M1-70, Biolegend), anti–CD11c BV421 (clone N418, Biolegend), anti–CD3 PE-Cy7 (clone 145-2C11, Biolegend), anti–CD4 Alexa Fluor 700 (clone GK1.5, eBioscience), anti–CD8 PerCP/Cy5.5 (clone 53-6.7, Biolegend), and anti–CD25 PE-Cy5 (clone PC61.5, Invitrogen). Tregs were stained by anti–FoxP3 APC (clone FJK-16s, Invitrogen) with the FoxP3/transcription factor–staining buffer set (eBioscience, 00-5523-00). Stained cells were fixed (Biolegend, 420801) and stored in the dark at 4°C for 24 to 48 hours. Samples were analyzed with a BD LSR II flow cytometer. Data analysis was performed using FlowJo, version 10.6.1 (Becton, Dickinson and Company). Fluorescence minus 1 (FMO) controls were used to set the boundary gates between positive and negative populations. Samples with <65% viable cells were excluded from analysis.

### Quantitative RT-PCR.

As previously published (31, 63), terminal tumor tissue was stored in RNA Later (AM7020, Thermo Fisher Scientific) at –20 °C. Tumors were homogenized in liquid nitrogen and resuspended in Trizol (Thermo Fisher Scientific, 15596018) for subsequent chloroform RNA extraction. For KRIMS-1 samples, cells were cultured in 12-well plates and treated with DMSO, Gem 15 nM, DAC 128 nM, or Gem + DAC using the dosing scheme in Figure 3A. On treatment day 4, cells were trypsinized and centrifuged at 500*g* for 5 minutes. The supernatant was removed and 1 mL of Trizol was added. RNA was isolated using the Direct-zol RNA MiniPrep (Zymo Research, R2052). cDNA was synthesized from 1 μg of RNA using iScript (Bio-Rad, 1708891). Reverse transcription–quantitative PCR was performed with PowerUp Sybr Green 2X Master Mix (Thermo Fisher Scientific, A25778) per the manufacturer’s instructions on an Applied Biosystems 7900HT instrument using the comparative Ct relative to 18s rRNA expression (tumor tissue) or B2m expression (KRIMS-1 cells) (Genomics Division of the Iowa Institute of Human Genetics, University of Iowa). Primer sequences are listed in Supplemental Table 2.

### Cell viability and synergy assays.

KRIMS-1 cells were previously developed in author RDD’s laboratory (31) and were grown in 10 cm dishes maintained in DMEM media containing 10% FBS, 1% penicillin-streptomycin (Pen-Strep, Gibco, 15140-122), and 1 mM sodium pyruvate (Gibco, 11360-070). For cell viability assays, cells were plated on day 0 in a 96-well (1.6 × 10^3^ cells/well) or 12-well (1.6 × 10^4^ cells/well) plate. After 24 hours (day 1), cells were treated with either Gem hydrochloride (MedChemExpress, HY-B0003; Sigma-Aldrich, 504594) diluted in water, 2′-deoxythymidine diluted in DMEM (Sigma-Aldrich, T1895-1G), or media control. On day 2, all media were removed and replaced with media containing either DAC (Selleckchem, S1200) diluted in DMSO or an equivalent concentration of DMSO. For rescue experiments, uridine (Sigma-Aldrich, U3003-5G) or 2′-deoxycytidine hydrochloride (MedChemExpress, HY-17564) diluted in DMEM was added during DAC treatment. This was repeated on day 3. On day 4, resazurin (Sigma-Aldrich, R7017) dissolved in PBS (1.5 mg/mL) was added to wells (20 μL for 96-well plates, 200 μL for 12-well plates) and cells were returned to the tissue culture incubator for 1 to 2 hours before being read on a microplate reader (BioTek). Fluorescence was normalized to DMSO-treated cells to determine percent viability. Drug interactions were analyzed using SynergyFinder 2.0 (33). Human cells lines were provided by the laboratories of author DJG, Michael Henry (University of Iowa, Iowa City, Iowa, USA), and Munir Tanas (University of Iowa, Iowa City, Iowa, USA). Human cell lines were maintained as follows: A673 and RD were cultured in DMEM with 10% FBS, 1% penicillin-streptomycin, and 1% sodium pyruvate; SJRH30 was cultured in RPMI with 10% FBS, 1% penicillin-streptomycin, and 1% sodium pyruvate; sNF96.2 was cultured in DMEM with 10% FBS, 1% penicillin-streptomycin, and 2 mM l-glutamate (Gibco, 25030-081); MIA PaCa-2 and PANC-1 were cultured in DMEM with 10% FBS and 1× MEM nonessential amino acids (Gibco, 111140-050); OV-90 was cultured in Medium 199 with 10% FBS and 1× MEM nonessential amino acids; and RT4 was cultured in McCoy’s 5A Medium with 10% FBS and 1× MEM nonessential amino acids. Cells from the following cell lines were plated in 96-well plates and treated and analyzed as described above: A673 (1.7 × 10^3^ cells/well), RD (5.6 × 10^3^ cells/well), SJRH30 (3.1 × 10^3^ cells/well), sNF96.2 (7.9 × 10^3^ cells/well), MIA PaCa-2 (2.5 × 10^3^ cells/well), PANC-1 (3 × 10^3^ cells/well), OV-90 (2 × 10^3^ cells/well), and RT4 (4 × 10^3^ cells/well). Drug treatments in human cell lines were performed in their corresponding media. For longitudinal proliferation assays, KRIMS-1 cells were plated in 12-well plates on day 0 and treated as described above. The resazurin viability assay was performed on days 1, 2, 3, and 4. Measurements were normalized to each treatment’s respective day 1 value to determine longitudinal fold change.

### Cell morphology, cell cycle, and apoptosis analysis.

KRIMS-1 cells were cultured in 12-well plates and treated with DMSO, Gem 15 nM, DAC 128 nM, or Gem + DAC using the dosing scheme in Figure 3A. On day 4, brightfield images of cells were take using an EVOS XL Core imaging system (Invitrogen) (Supplemental Figure 11, A–E; 40× magnification; scale bars,100 μm). After imaging, cells were stained with EdU and propidium iodide (Invitrogen, C10420) or with annexin V and propidium iodide (Biolegend, 640932) according to the manufacturer’s instructions. For annexin V staining, detached cells were isolated from the media by centrifugation (500*g* for 5 minutes) and combined with trypsinized adhered cells before proceeding with the standard staining protocol. Samples were analyzed with a BD LSR II flow cytometer. Data analysis was performed using FlowJo, version 10.6.1 (Becton, Dickinson and Company).

### Western blots.

For cell lysate preparation, KRIMS-1 cells were cultured in 12-well plates and treated with DMSO, Gem 15 nM, DAC 128 nM, or Gem + DAC using the dosing scheme in Figure 3A or with camptothecin 20 μM (Sigma-Aldrich, 208925) for 24 hours. On day 4, cells were washed twice with 1× PBS, then lysed in 1× Laemmli sample buffer (Bio-Rad, 1610747) prepared according to the manufacturer’s instructions. Lysates were heated to 95°C for 5 to 10 minutes, then sheared with a 25G needle and syringe. Lysates were centrifuged at 16,000*g* for 20 minutes at room temperature. Supernatants were transferred to new tubes and stored at 4°C. Protein quantification was performed using Pierce 660 nM Protein Assay Reagent (Thermo Scientific, 22660) and neutralizer (G Biosciences, 786-604) according to manufacturers’ instructions. Equal amounts of protein were loaded in NuPAGE 4 to 12% Bis-Tris gels (Invitrogen, NP0335/NP0336). Gels were run at 50V for 20 minutes and 120V for 90 minutes using NuPAGE MES SDS Running Buffer (Novex, NP0002). Samples were transferred to PVDF (Millipore, IPFL00010) at 20V for 1 hour in NuPAGE Transfer Buffer (Novex, NP0006-1). Blots were blocked in 10% milk (1 g of powdered milk in 10 mL Tris Buffered Saline (Bio-Rad, 1706435) with 0.1% Tween [TBS-T]) for 1 hour at room temperature. Blots were rinsed with TBS-T, then incubated in primary Ab in TBS-T for 24 to 72 hours at 4°C. Blots were washed in TBS-T 3 times for 10 minutes each, then incubated in HRP-conjugated secondary Ab in TBS-T for 1 hour at room temperature. Blots were again washed in TBS-T 3 times for 10 minutes each. Blots were imaged using a ChemiDoc (Bio-Rad) and SuperSignal West Pico PLUS Chemiluminescent Substrate (Thermo Scientific, 34577). Densitometry measurements were taken using Fiji software (64). The primary Abs used were anti-DNMT1 (Cell Signaling Technology, 5032S, 1:1000), anti-PARP (Cell Signaling Technology, 9532S, 1:1000), and anti–γ-tubulin (Sigma-Aldrich, T5326, 1:10,000). The secondary Abs used were goat anti–rabbit HRP and goat anti–mouse HRP (Jackson ImmunoResearch Laboratories, 111-035-144, 115-035-146).

### DNA isolation and 5-methylcytosine dot blot.

Genomic DNA was isolated using a GeneElute Mammalian Genomic DNA Minipret Kit (Sigma-Aldrich, G1N350-1KT) from KRIMS-1 cells cultured in 12-well plates and treated with DMSO, Gem 15 nM, DAC 128 nM, or Gem + DAC using the dosing scheme in Figure 3A. DNA was eluted in nuclease-free water. We combined 25 μL of DNA (100 ng/μL) with 25 μL of 2× DNA denaturing buffer (200 mM NaOH and 20 mM EDTA in water), heated to 95°C for 10 minutes, combined with 50 μL of 20× saline sodium citrate (3.0 M NaCl and 0.3 M sodium citrate in water with a final pH of 7.0), and immediately chilled on ice for 5 minutes. Next, 25 μL of nuclease-free water was added for a final concentration of 20 ng/μL of DNA. A series of six 2-fold dilutions were made, generating 7 dilutions of the DNA ranging from 20 ng/μL to 0.3125 ng/μL. An eighth sample containing only nuclease-free water (no DNA) was also created.

For the dot blot, positively charged nylon membranes (Roche, 11209299001) were briefly wet with 10× saline sodium citrate buffer, placed on the dot-blot apparatus, and gently dried with vacuum pressure before adding 50 μL of each sample with vacuum pressure. The membrane was plastic wrapped and UV cross-linked at 1200 J/m^2^. Blots were blocked in 5% milk (0.5 g of powdered milk in 10 mL Tris Buffered Saline (Bio-Rad, 1706435) with 0.1% TBS-T) for 1 hour at room temperature, rinsed with TBS-T, and incubated in primary Ab in TBS-T for 24 hours at 4°C. Blots were washed in TBS-T 3 times for 10 minutes each, incubated in HRP-conjugated secondary Ab in TBS-T for 1 hour at room temperature and washed in TBS-T 3 times for 10 minutes each. Blots were imaged using a ChemiDoc (Bio-Rad) and SuperSignal West Pico PLUS Chemiluminescent Substrate (Thermo Scientific, 34577). After imaging, blots were washed in TBS-T for 5 minutes, then in 100% ethanol (Decon Laboratories, 2701) for 2 minutes, and finally in water for 2 minutes. Blots were then stained with 0.2% methylene blue (Fisher Scientific, M291-25) in 0.3 M sodium acetate (Research Products International, S22045-500.0) for 5 minutes, rinsed with water, and imaged using a ChemiDoc (Bio-Rad). The primary Ab was anti–5-methylcytosine (Cell Signaling Technology, 28692S, 1:2000); the secondary Ab was anti–rabbit HRP (Jackson ImmunoResearch Laboratories, 111-035-144).

### Statistics.

Statistical analysis was performed using GraphPad Prism 8. A *P* value <0.05 was considered significant. In vivo data with 3 or more groups were analyzed with Welch’s ANOVA and Dunnett’s T3 multiple comparison test. In vitro data were analyzed using unpaired *t* tests with Welch’s correction (comparisons with 2 groups) or ordinary 1-way ANOVA and Tukey’s multiple comparison test (comparisons with 3 or more groups). Survival curves were analyzed by log-rank (Mantel–Cox) test.

### Study approval.

All animal procedures for this study were approved by the IACUC of the University of Iowa and were carried out in accordance with Animal Research: Reporting of In Vivo Experiments guidelines.

## Author contributions

RDD, WRG, and VM conceived and designed the study. WRG, AS, JDR, EAL, APS, GRM, QRB, VKA, and GAR performed the experiments. WRG collected and analyzed the data. RDD, WRG, AS, DEQ, and DJG interpreted the data. WRG and RDD wrote the paper. RDD acquired the funding and supervised the study. All authors reviewed and approved the manuscript.

## Supplementary Material

Supplemental data

## Version 1 10/13/2022

In-Press Preview

## Version 2 11/22/2022

Electronic publication
