# Supplementary material for: Augmenting chemotherapy with low-dose decitabine through an immune-independent mechanism
Source: JCI Insight. 2022 Nov 22;7(22):e159419. doi: 10.1172/jci.insight.159419 (PMC9746804; doi:10.1172/jci.insight.159419)
Supplement: Supplemental data [file jciinsight-7-159419-s128.pdf]

Title: Augmenting chemotherapy with low-dose decitabine through an immune-independent mechanism

Wade R. Gutierrez [1,2,3,4], Amanda Scherer [3,4], Jeffrey D. Rytlewski [4], Emily A. Laverty [4], Alexa P. Sheehan [3,4,6], Gavin R. McGivney [1,3,4,5], Qierra R. Brockman [3,4,6], Vickie Knepper-Adrian [4], Grace A. Roughton [4], Dawn E. Quelle [1,2,3,6,7,8], David J. Gordon [3,9], Varun Monga [3,4], and Rebecca D. Dodd [1,2,3,4,6]

[1] Cancer Biology Graduate Program, University of Iowa, Iowa City, Iowa, USA

[2] Medical Scientist Training Program, University of Iowa, Iowa City, Iowa, USA

[3] Holden Comprehensive Cancer Center, University of Iowa, Iowa City, Iowa, USA

[4] Department of Internal Medicine, University of Iowa, Iowa City, Iowa, USA

[5] Department of Molecular Physiology and Biophysics, University of Iowa, Iowa City, Iowa, USA

[6] Molecular Medicine Graduate Program, University of Iowa, Iowa City, Iowa, USA

[7] Department of Neuroscience and Pharmacology, University of Iowa, Iowa City, Iowa, USA

[8] Department of Pathology, University of Iowa, Iowa City, Iowa, USA

[9] Department of Pediatrics, University of Iowa, Iowa City, Iowa, USA

Corresponding author:

Rebecca D. Dodd

Carver College of Medicine, University of Iowa, 375 Newton Rd, 5206 MERF, Iowa City, Iowa 52246

Tel: 319-335-4962, Email: rebecca-dodd@uiowa.edu

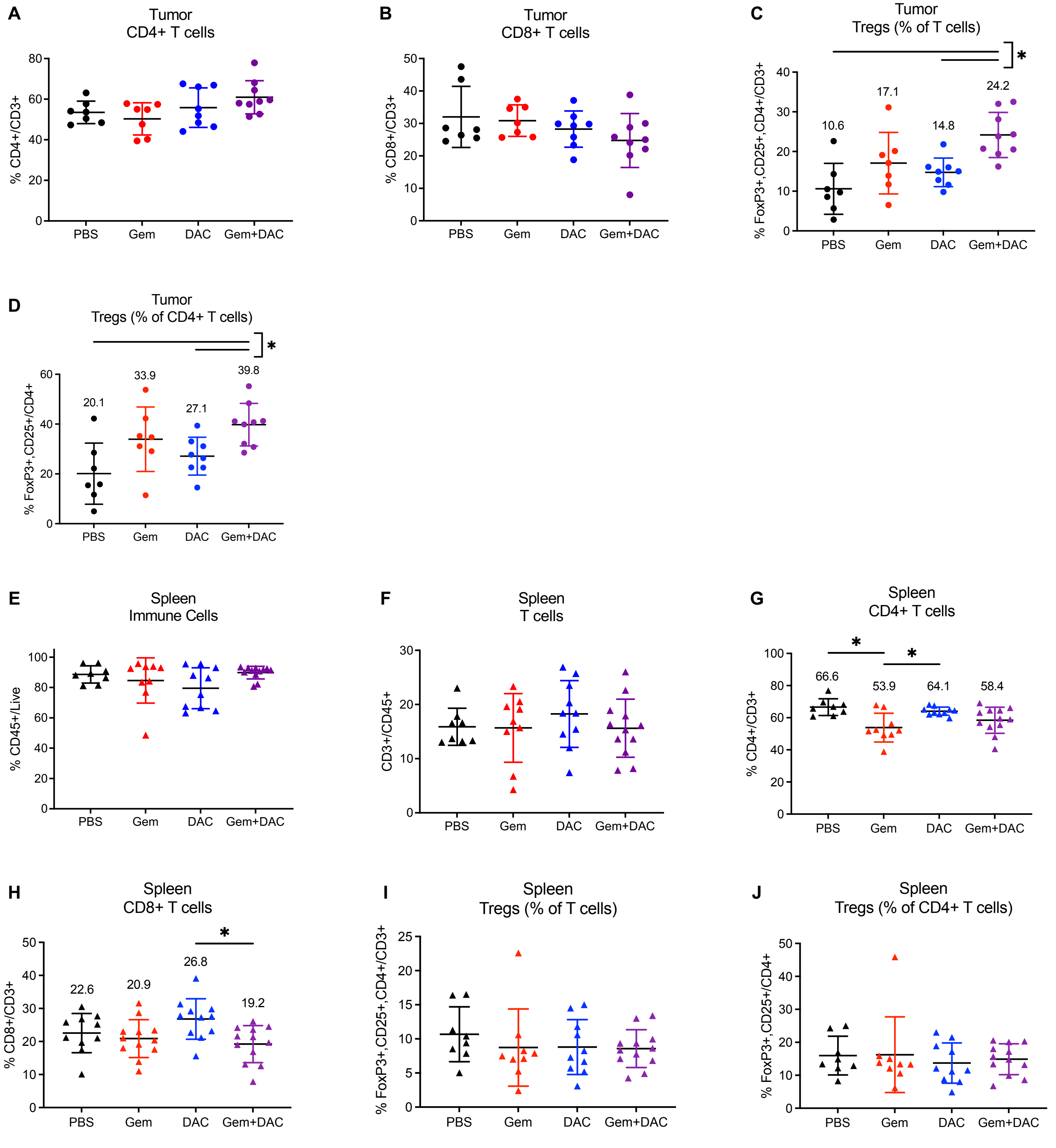

Supplemental Figure 1: KP UPS tumor and spleen immune populations. (**A-D**) T cell subsets in KP UPS tumors (*n* = 7-9/group). (**E-J**) Immune cells and T cell subsets in spleens from mice with KP UPS tumors (*n* = 7-9/group, same mice as in A-D). Data represent the mean ± SD. Welch's ANOVA and Dunnett's T3 multiple comparison test used to analyze A-J. *$P < 0.05$.

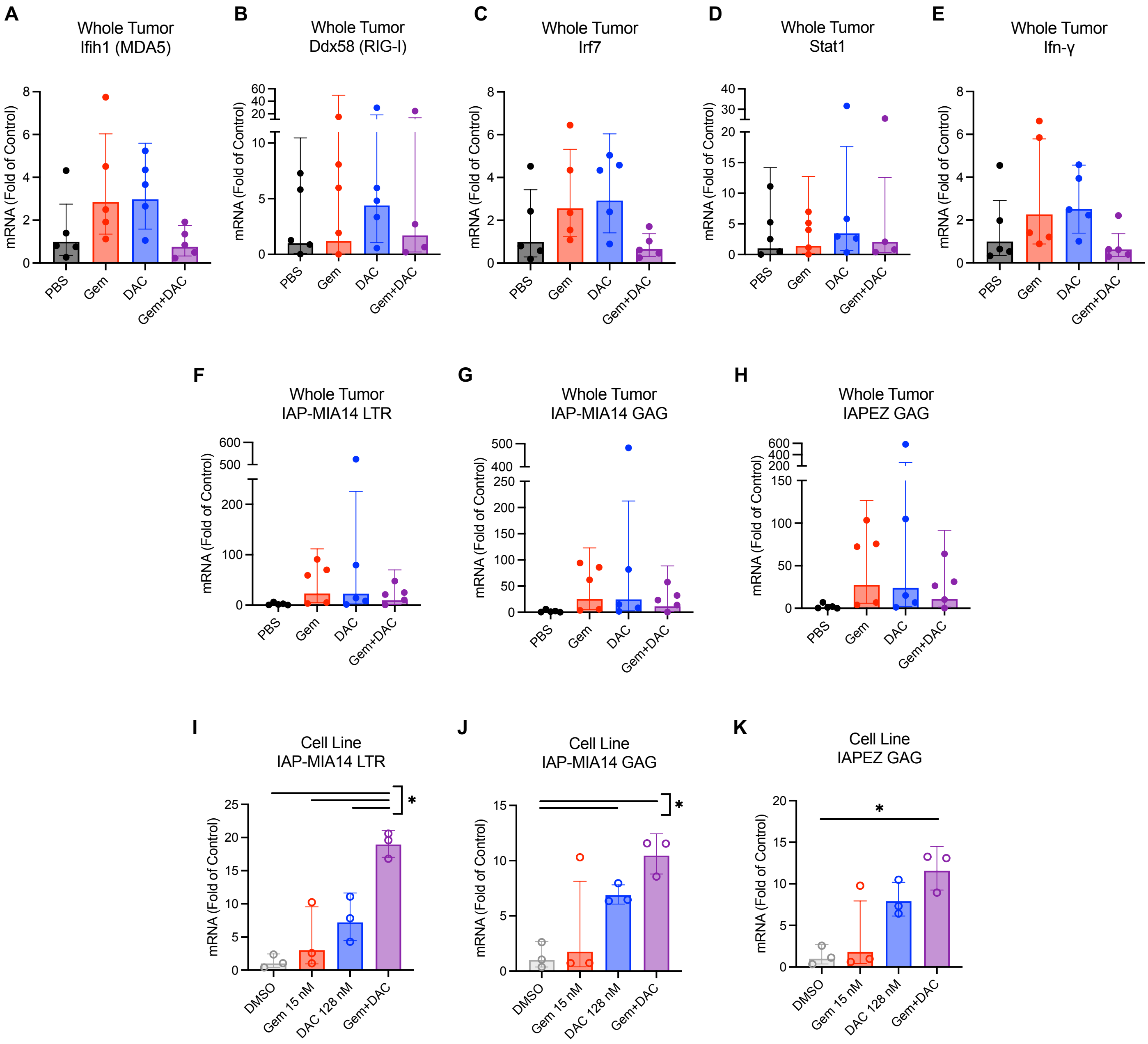

<u>Supplemental Figure 2: Viral response pathway and endogenous retrovirus gene expression.</u> (**A**-**E**) RNA was isolated from KP UPS tumors and levels of key viral response pathway genes were assessed by qPCR (*n* = 5 mice per group). (**F**-**H**) Expression of endogenous retroviral (ERV) transcripts in KP UPS tumors (*n* = 5 mice per group). (**I**-**K**) Expression of ERV transcripts in KRIMS-1 cells treated using the dosing scheme in Figure 3A. Data represent independent experiments (*n* = 3). Welch's ANOVA and Dunnett's T3 multiple comparison test used to analyze A-H. Ordinary one-way ANOVA and Tukey's multiple comparisons test used to analyze I-K. **P* < 0.05.

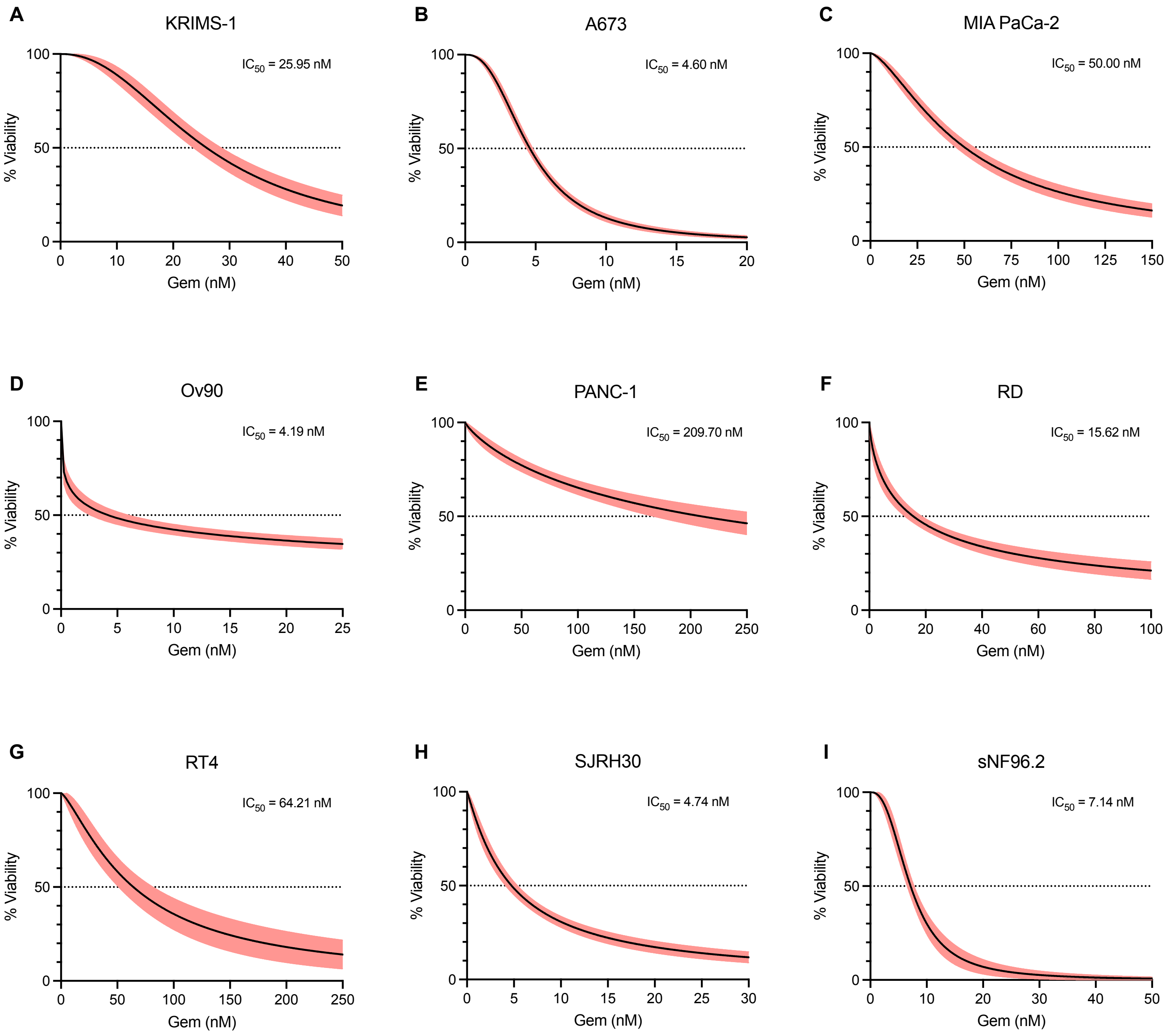

<u>Supplemental Figure 3: Gemcitabine IC_{50} curves.</u> (**A-I**) Gemcitabine IC_{50} curves for KRIMS-1 and human cell lines. Cells treated using the single-agent treatment scheme in Figure 3A. Cell viability was measured on Day 4 using a resazurin assay. Data from two or three independent experiments included in each plot. Curves fitted using nonlinear regression, option “[inhibitor] vs normalized response-variable slope” in GraphPad Prism. Shaded areas represent 95% confidence intervals.

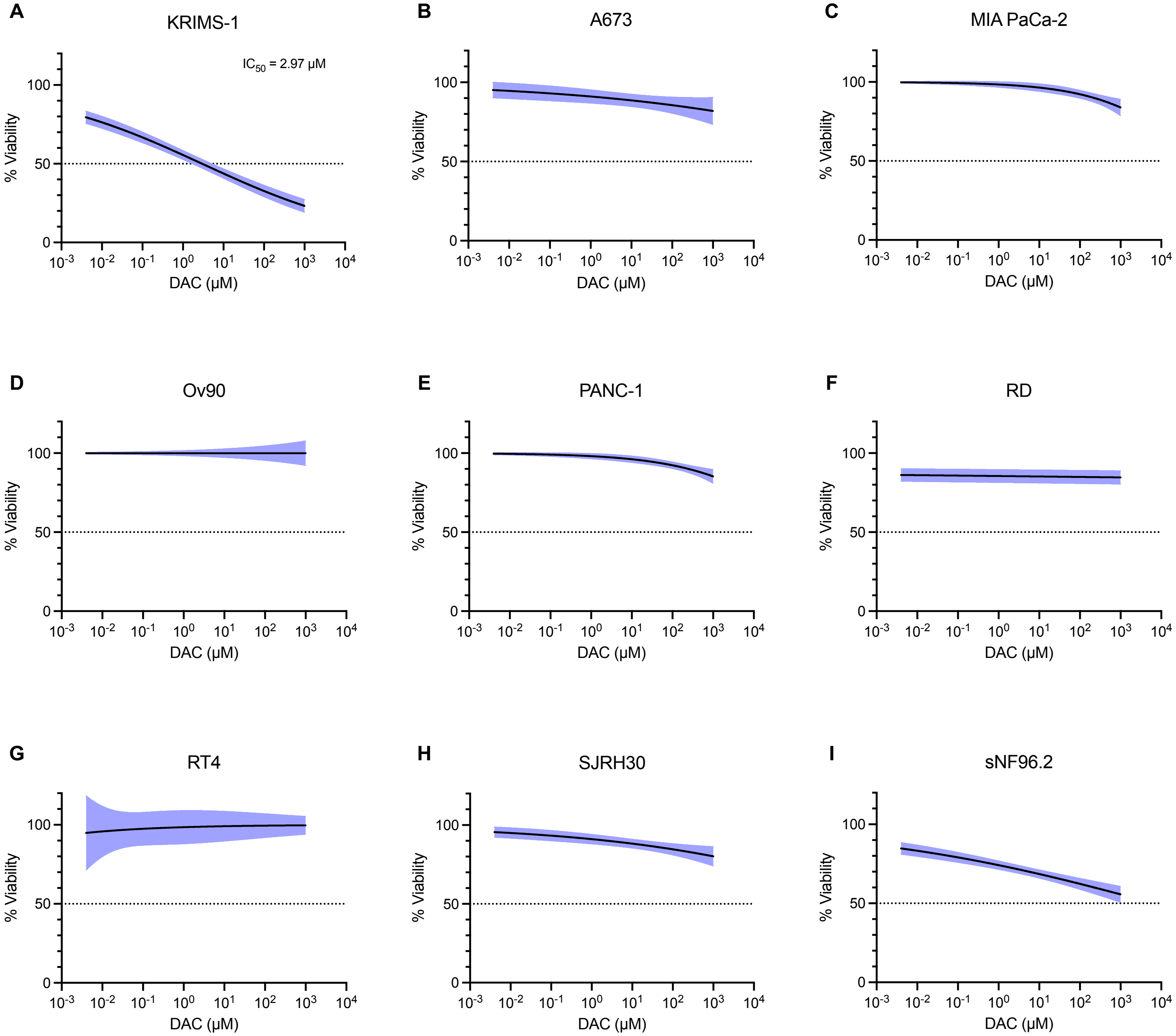

Supplemental Figure 4: Decitabine IC_{50} curves. (**A-I**) Decitabine IC_{50} curves for KRIMS-1 and human cell lines. Cells treated using the single-agent treatment scheme in Figure 3A. Cell viability was measured on Day 4 using a resazurin assay. Data from two or three independent experiments included in each plot. Curves fitted using nonlinear regression, option "[inhibitor] vs normalized response-variable slope" in GraphPad Prism. Shaded areas represent 95% confidence intervals.

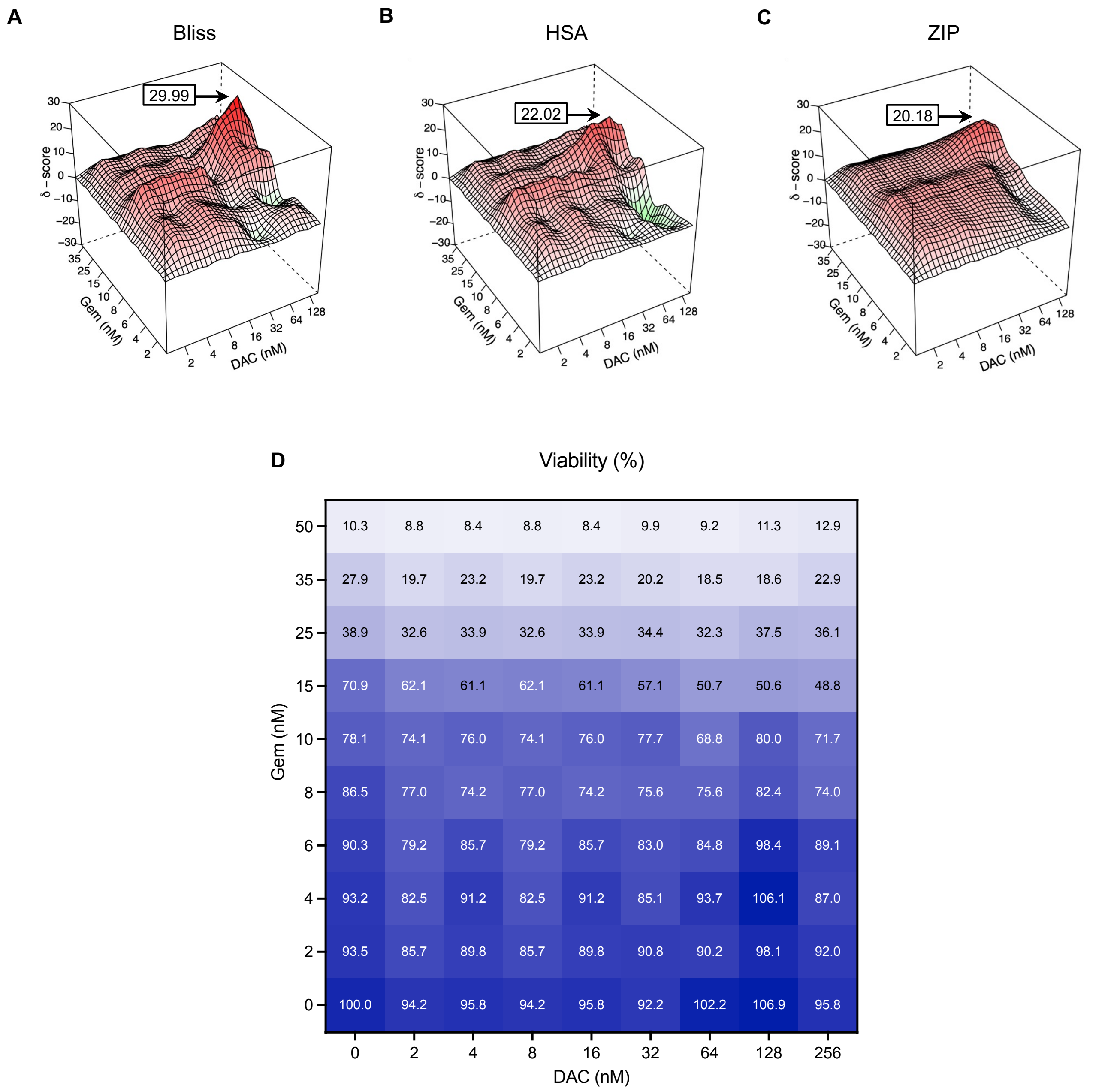

<u>Supplemental Figure 5: Synergy analyses and percent inhibition.</u> (**A**-**C**) Analysis of data from Figure 3B using three different synergy analysis methods: Bliss independence (Bliss), highest single agent (HSA), and zero interaction potency (ZIP). Gem+DAC is generally additive (δ-score between 0 and 10), with a strong synergistic interaction (δ-score greater than 10) occurring with 15 nM gemcitabine and 128 nM decitabine. (**D**) Raw viability data analyzed in A-C. Values are the average of three technical replicates. Data in A-D are from one representative experiment performed in quadruplicate.

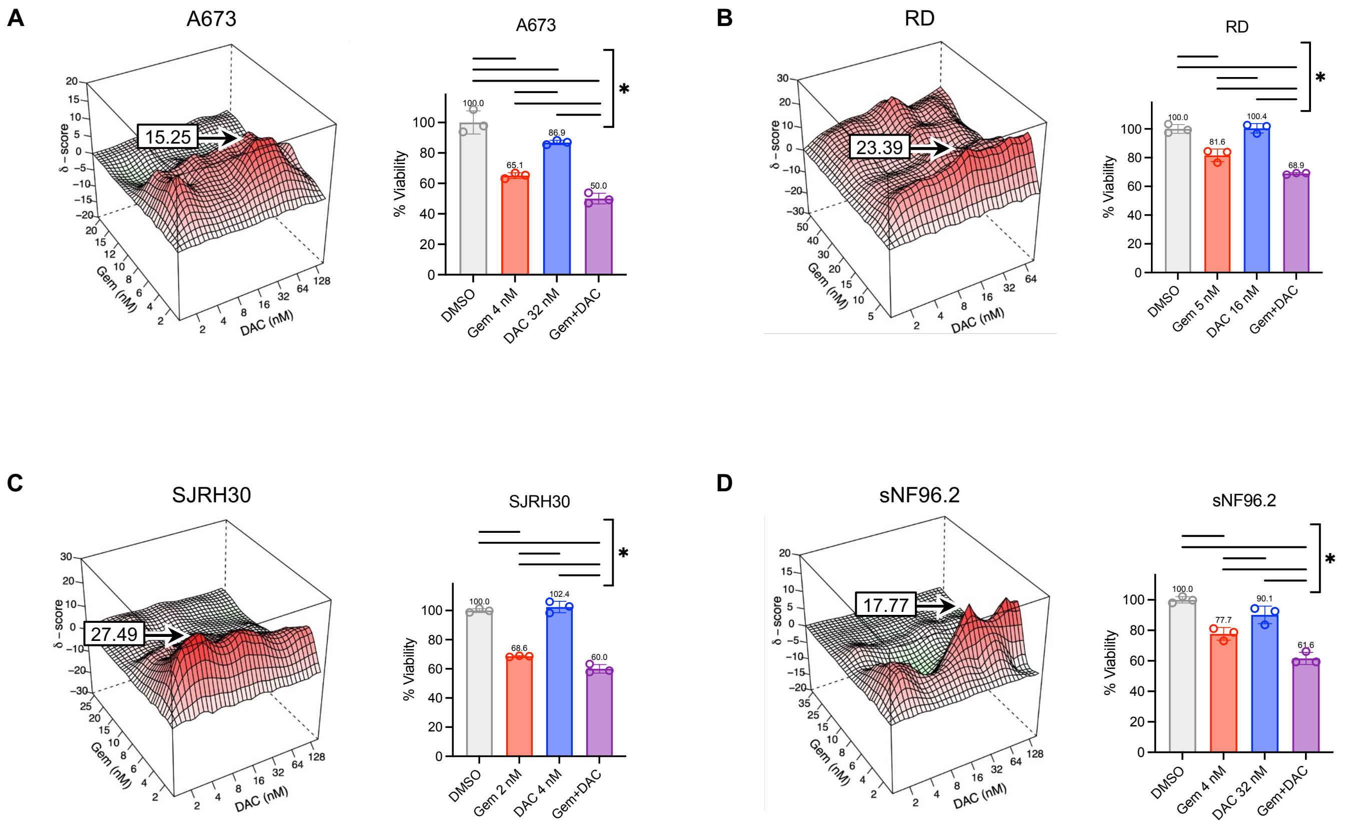

Supplemental Figure 6: Gem+DAC efficacy in human sarcoma cell lines. (**A-D**) Human sarcoma cell lines A673 (Ewing's sarcoma, A), RD (embryonal rhabdomyosarcoma, B), SJRH30 (alveolar rhabdomyosarcoma, C), and sNF96.2 (malignant peripheral nerve sheath tumor, D), were treated using the dosing scheme in Figure 3A. Cell viability was measured on Day 4 using a resazurin assay. Bliss synergy analysis was performed, and the area of greatest synergy was quantified. Synergy plots and bar graphs are representative. Data represent technical replicates and the mean ± SD. Ordinary one-way ANOVA and Tukey's multiple comparisons test used for analysis. *$P < 0.05$.

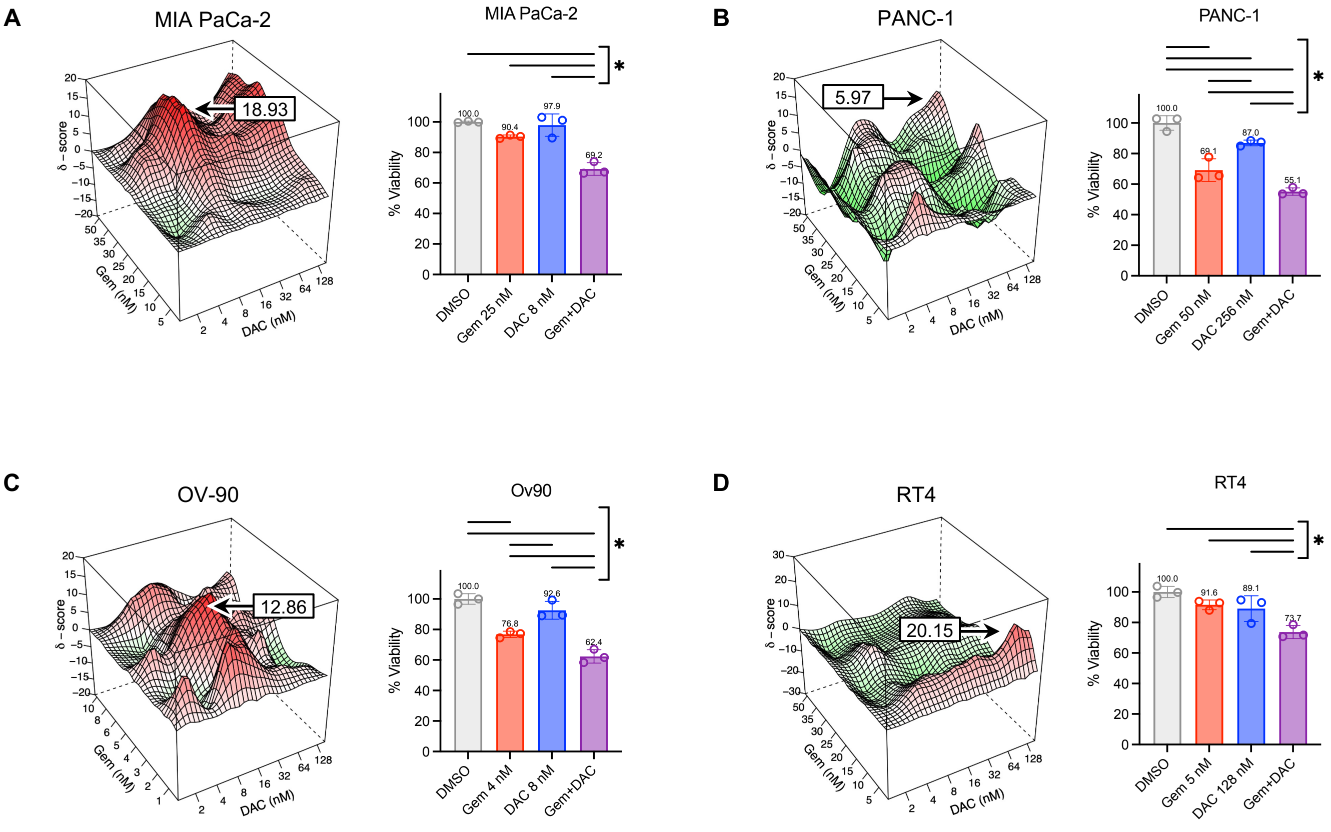

Supplemental Figure 7: Gem+DAC efficacy in human carcinoma cell lines. (**A**-**D**) Human carcinoma cell lines MIA PaCa-2 (pancreatic ductal adenocarcinoma, A), PANC-1 (pancreatic ductal adenocarcinoma, B), Ov90 (ovarian cancer, C), and RT4 (non-muscle invasive bladder cancer, D), were treated using the dosing scheme in Figure 3A. Cell viability was measured on Day 4 using a resazurin assay. Bliss synergy analysis was performed, and the area of greatest synergy was quantified. Synergy plots and bar graphs are representative. Data represent technical replicates and the mean ± SD. Ordinary one-way ANOVA and Tukey's multiple comparisons test used for analysis. *$P < 0.05$.

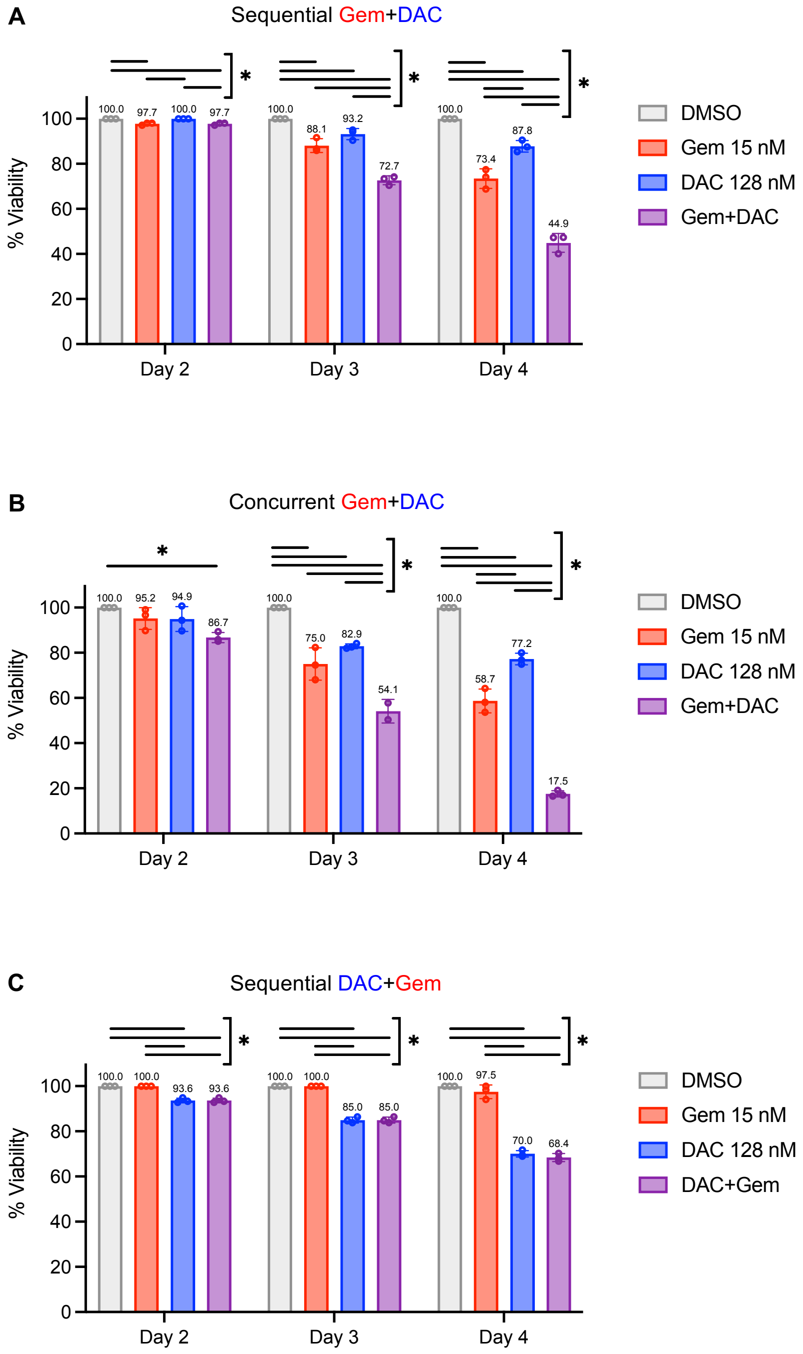

Supplemental Figure 8: Longitudinal sequence-dependent viability. (**A-C**) Individual viability measurements used in Figure 3C-H. Data represent independent experiments (n = 3) and the mean ± SEM. Ordinary one-way ANOVA and Tukey's multiple comparisons test used for analysis. $^*P < 0.05$.

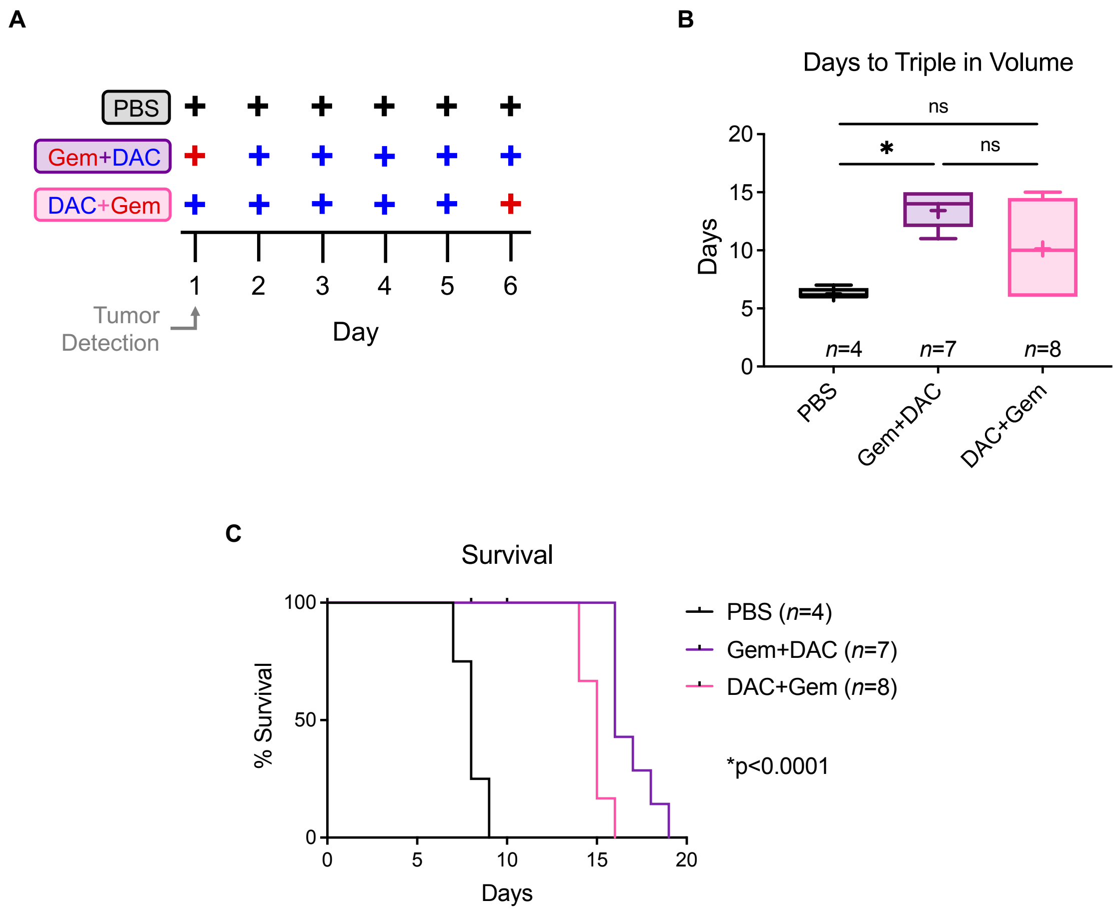

Supplemental Figure 9: Effect of drug sequence in vivo. (**A**) At the time of tumor detection, mice enrolled in one of four experimental arms: six doses of PBS, one dose of gemcitabine (150 mg/kg) followed by five doses of decitabine (0.2 mg/kg), or five doses of decitabine (0.2 mg/kg) followed by one dose of gemcitabine (150 mg/kg). (**B**) Treatment with Gem+DAC but not DAC+Gem significantly slowed tumor growth compared to PBS. Growth rates are reported as time required for tumors to triple in volume (n = 4-8/group). Boxes represent 25th and 75th percentiles. Whiskers represent minimum and maximum values. Horizonal line represents median; + represents mean. (**C**) Both Gem+DAC and DAC+Gem extended survival compared to PBS. Gem+DAC extended survival significantly more than DAC+Gem. Welch's ANOVA and Dunnett's T3 multiple comparison test used to analyze B. Log-rank (Mantel-Cox) tests used to analyze C. *$P < 0.05$.

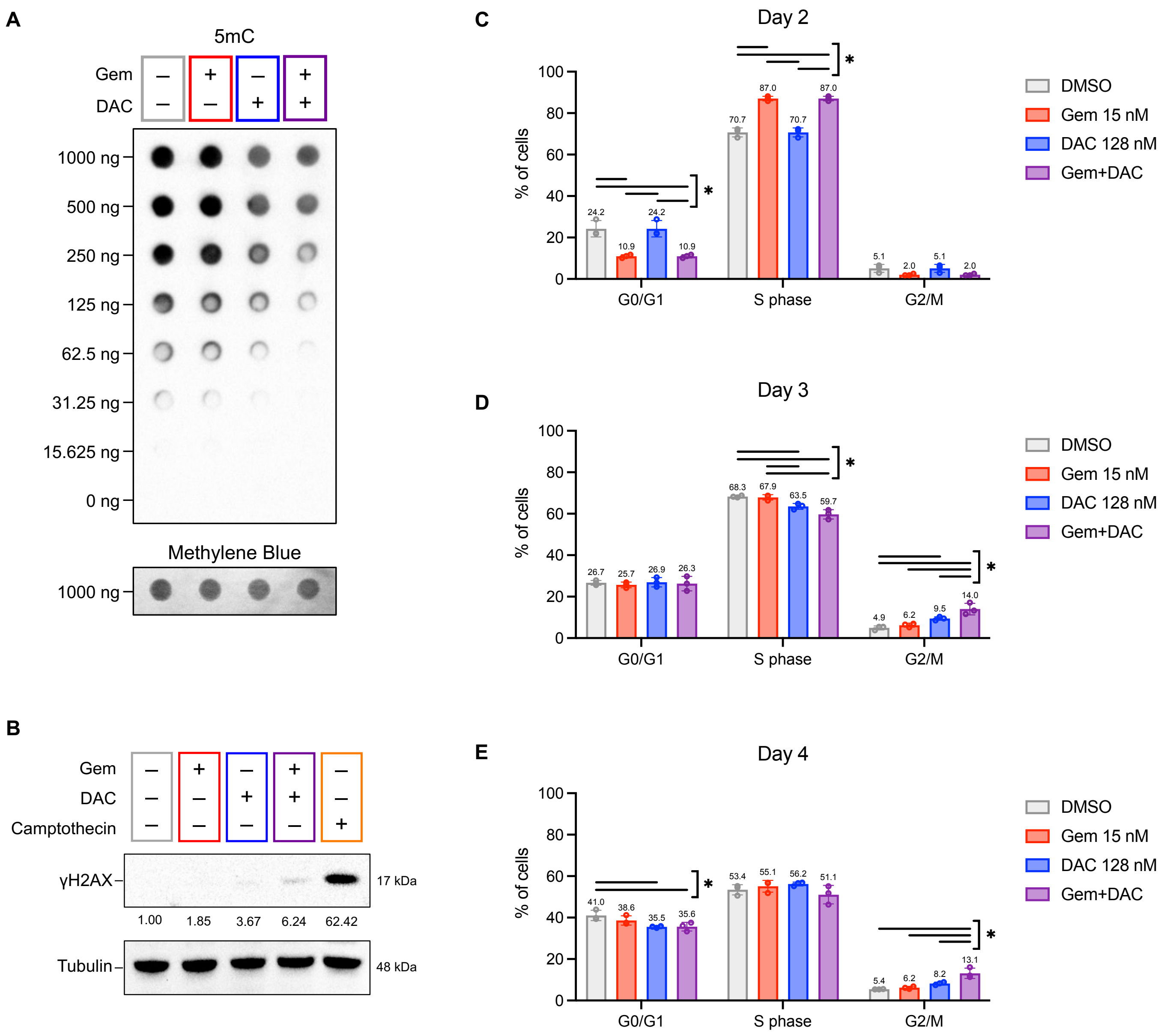

Supplemental Figure 10: 5mC dot blot, γH2AX western blot and Gem+DAC longitudinal cell cycle analysis. (**A**) Representative 5-methylcytosine dot blot using genomic DNA from cells collected on day 4. Methylene blue staining used as loading control. (**B**) Representative western of blot lysates collected on day 4. Gem+DAC leads to only modestly induces γH2AX when compared to cells treated with camptothecin, a topoisomerase inhibitor and potent DNA damaging agent. (**C-E**) Complete statistical analysis of Figure 4F. Analysis performed on day 2 (A), day 3 (B), and day 4 (C). Data represent independent experiments (*n* = 3) and the mean ± SEM. Ordinary one-way ANOVA and Tukey's multiple comparisons test used for analysis. $^*P < 0.05$.

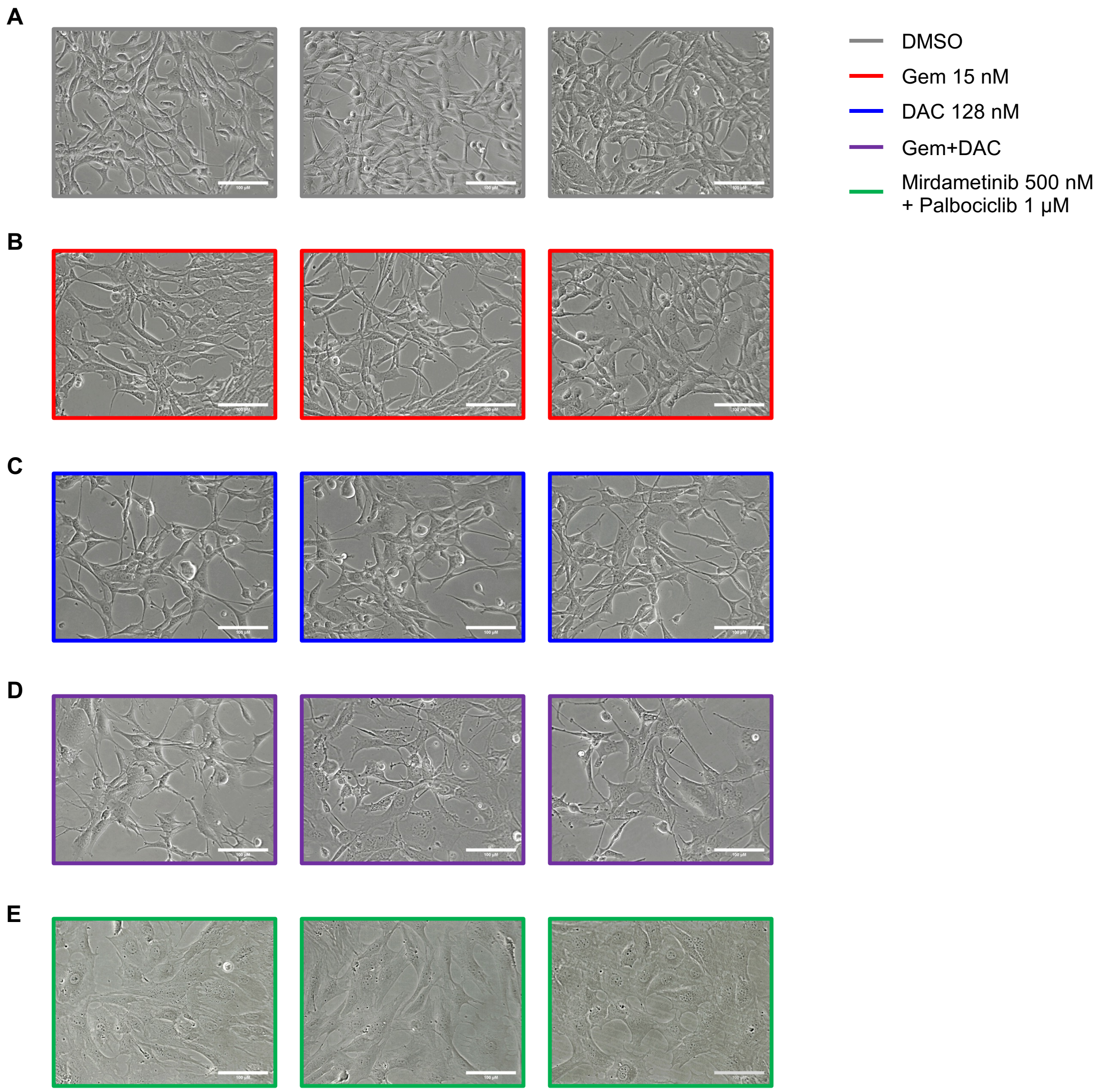

<u>Supplemental Figure 11: Gem+DAC effects on cell morphology.</u> (**A**-**E**) Bright field microscopy images of KRIMS-1 cells treated with dosing scheme in Figure 3A or with Mirdametinib+Palbociclib (MEK and CDK4/6 inhibitor) on days 1-3. Gem+DAC and single agent controls cause only modest senescence phenotypic changes (cell flattening, enlarged nuclei) compared the Mirdametinib+Palbociclib positive control. Images are representative. All images taken on day 4 from the same experiment. Scale bars: 100 μm.

A

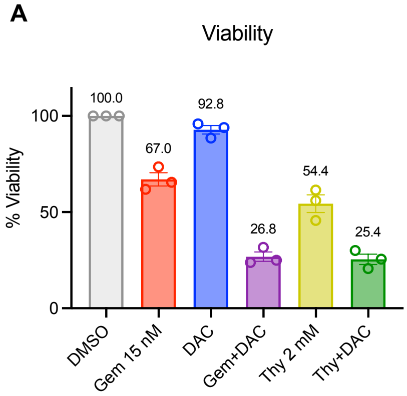

	PBS	Gem	DAC	Gem+DAC	Thy	Thy+DAC
PBS		*	ns	*	*	*
Gem	*		*	*	ns	*
DAC	ns	*		*	*	*
Gem+DAC	*	*	*		*	ns
Thy	*	ns	*	*		*
Thy+DAC	*	*	*	ns	*	

B

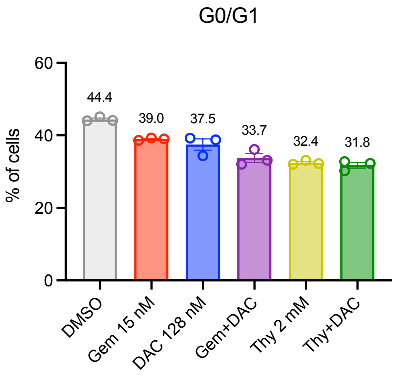

	DMSO	Gem	DAC	Gem+DAC	Thy	Thy+DAC
DMSO		ns	*	*	*	*
Gem	ns		ns	*	*	*
DAC	*	ns		*	ns	*
Gem+DAC	*	*	*		ns	ns
Thy	*	*	ns	ns		*
Thy+DAC	*	*	*	ns	*	

C

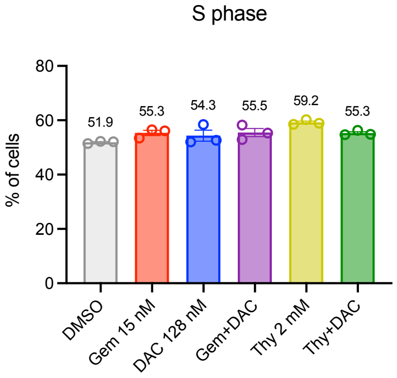

	DMSO	Gem	DAC	Gem+DAC	Thy	Thy+DAC
DMSO		ns	ns	ns	*	ns
Gem	ns		ns	ns	ns	ns
DAC	ns	ns		ns	ns	ns
Gem+DAC	ns	ns	ns		ns	ns
Thy	*	ns	ns	ns		ns
Thy+DAC	ns	ns	ns	ns	ns	

D

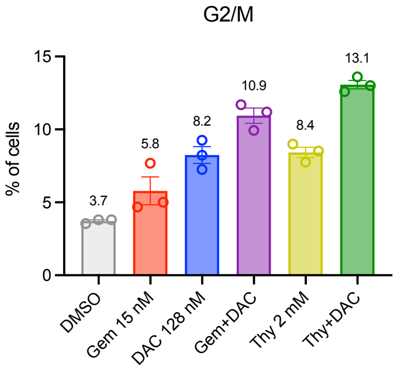

	DMSO	Gem	DAC	Gem+DAC	Thy	Thy+DAC
DMSO		*	*	*	*	*
Gem	*		ns	*	*	*
DAC	*	ns		ns	*	*
Gem+DAC	*	*	ns		ns	ns
Thy	*	*	*	ns		ns
Thy+DAC	*	*	*	ns	ns	

<u>Supplemental Figure 12: Gem+DAC and Thy+DAC viability and cell cycle analysis.</u> (**A-D**) Complete statistical analysis of Figure 5C-D. Data represent independent experiments (n = 3) and the mean ± SEM. Ordinary one-way ANOVA and Tukey's multiple comparisons test used for analysis. *$P < 0.05$.

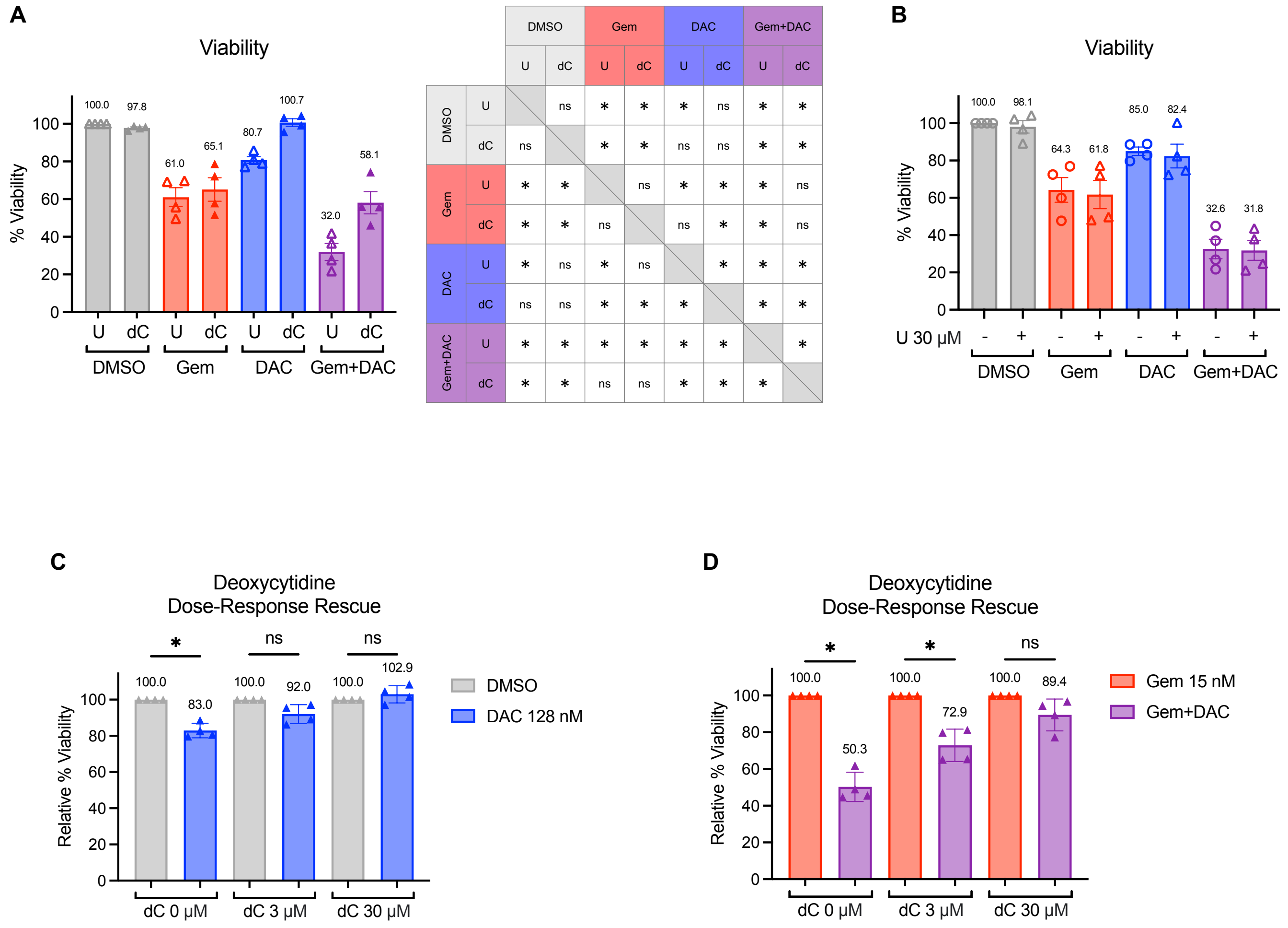

		DMSO		Gem		DAC		Gem+DAC	
		U	dC	U	dC	U	dC	U	dC
DMSO	U		ns	*	*	*	ns	*	*
	dC	ns		*	*	ns	ns	*	*
Gem	U	*	*		ns	*	*	*	ns
	dC	*	*	ns		ns	*	*	ns
DAC	U	*	ns	*	ns		*	*	*
	dC	ns	ns	*	*	*		*	*
Gem+DAC	U	*	*	*	*	*	*		*
	dC	*	*	ns	ns	*	*	*	

Supplemental Figure 13: Deoxycytidine (dC) viability rescue and dose-response. (**A**) Complete statistical analysis of Figure 5G. (**B**) Uridine supplementation did not impact treatment efficacy. (**C**) Relative day 4 viability of DAC+dC versus DMSO+dC. (**D**) Relative day 4 viability Gem+DAC+dC versus Gem+dC. Data represent independent experiments (n = 4) and the mean ± SEM. Ordinary one-way ANOVA and Tukey's multiple comparisons test used for analysis in A. Multiple unpaired t tests with Welch's correction used for analysis in B-D. *$P < 0.05$.

A

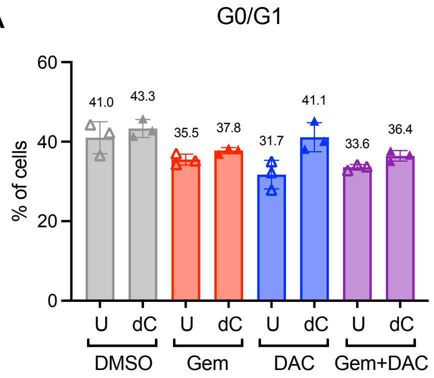

		DMSO U	DMSO dC	Gem U	Gem dC	DAC U	DAC dC	Gem+DAC U	Gem+DAC dC
DMSO	U		ns	ns	ns	*	ns	*	ns
	dC	ns		*	ns	*	ns	*	ns
Gem	U	ns	*		ns	ns	ns	ns	ns
	dC	ns	ns	ns		ns	ns	ns	ns
DAC	U	*	*	ns	ns		*	ns	ns
	dC	ns	ns	ns	ns	*		ns	ns
Gem+DAC	U	*	*	ns	ns	ns	ns		*
	dC	ns	ns	ns	ns	ns	ns	*	

B

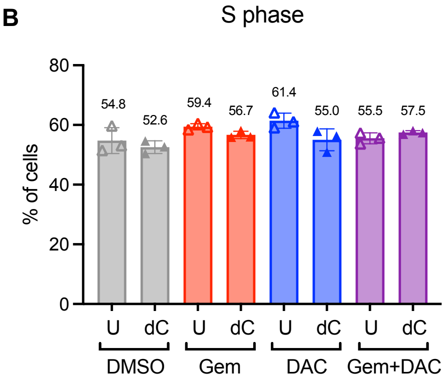

		DMSO U	DMSO dC	Gem U	Gem dC	DAC U	DAC dC	Gem+DAC U	Gem+DAC dC
DMSO	U		ns	ns	ns	ns	ns	ns	ns
	dC	ns		ns	ns	ns	ns	ns	ns
Gem	U	ns	ns		ns	ns	ns	ns	ns
	dC	ns	ns	ns		ns	ns	ns	ns
DAC	U	ns	ns	ns	ns		*	ns	ns
	dC	ns	ns	ns	ns	*		ns	ns
Gem+DAC	U	ns	ns	ns	ns	ns	ns		ns
	dC	ns	ns	ns	ns	ns	ns	ns	

C

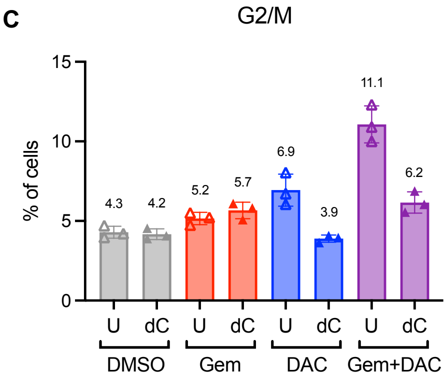

		DMSO U	DMSO dC	Gem U	Gem dC	DAC U	DAC dC	Gem+DAC U	Gem+DAC dC
DMSO	U		ns	ns	ns	*	ns	*	ns
	dC	ns		ns	ns	*	ns	*	*
Gem	U	ns	ns		ns	ns	ns	*	ns
	dC	ns	ns	ns		ns	ns	*	ns
DAC	U	*	*	ns	ns		*	*	ns
	dC	ns	ns	ns	ns	*		*	*
Gem+DAC	U	*	*	*	*	*	*		*
	dC	ns	*	ns	ns	ns	*	*	

Supplemental Figure 14: Deoxycytidine (dC) cell cycle rescue. (**A-C**) Complete statistical analysis of Figure 5H. Data represent independent experiments (n = 3) and the mean ± SEM. Ordinary one-way ANOVA and Tukey's multiple comparisons test used for analysis. *$P < 0.05$.

	Gem % viability	DAC % viability	Gem+DAC		
			Predicted additive response % viability	Actual % viability	p-value
Sequential Gem+DAC	73.4	87.8	61.2	44.9	0.0068*
Concurrent Gem+DAC	58.7	77.2	35.9	17.5	0.0152*
DAC+Gem	97.5	70.0	67.5	68.4	0.7179

Supplemental Table 1: Predicted vs actual Gem+DAC efficacy. The predicted additive response of Gem+DAC treatment was calculated using the formula: *Predicted additive response = 100% – (Gem viability decrease + DAC viability decrease)*. Sequential and concurrent Gem+DAC treatments reduced cell viability to a level significantly lower than the additive effect of monotherapy controls, demonstrating a synergistic interaction. DAC+Gem actual and predicted additive responses did not differ. Monotherapy and actual Gem+DAC viability values taken from Figure 3D, F, and H. Gem+DAC predicted additive response viability and actual viability compared using unpaired t test with Welch's correction.

Gene Name	Forward Primer Sequence	Reverse Primer Sequence
Ifih1 (MDA5)	GTGATGACGAGGCCAGCAGTTG	ATTCATCCGTTTCGTCCAGTTTCA
Ddx58 (RIG-I)	ACAAAGCGTGCTCAGTGTTT	CGTGGAAGAAGGCTTTGAGG
Irf7	CCTCTTGCTTCAGGTTCTGC	GGAGCCTGTGGTGGGAC
Stat1	GCTGTGCCTCTGGAATGATG	CGGGAGCTCTCACTGAATCT
Ifn-γ	TCTGGAGGAACTGGCAAAAG	TTCAAAGACTTCAAAGAGTCTGAGG
IAP-MIA14 LTR	GACACGTCCTAGGCGAAATATAAC	TATTGCTTACATCTTCAGGAGCAAG
IAP-MIA14 GAG	GATCAATTAGCGGAGGTCTCTAG	CCAGTCTGTTTCTTCAGAGGAGAA
IAPEZ GAG	GCTCTCCCTAGTATGGGCAAATAT	AATCTCTCTGCTCTGGAGTCAAAG
18s	GAGGCCCTGTAATTGGAATGA	GCAGCAACTTTAATATACGCTATTGG
B2m	GGTCTTTCTGGTGCTTGTCTC	GTTCAGTATGTTCGGCTTCCC

Supplemental Table 2: List of PCR Primers.
